# Formation of Si/SiO_2_ Luminescent Quantum Dots From Mesoporous Silicon by Sodium Tetraborate/Citric Acid Oxidation Treatment

**DOI:** 10.3389/fchem.2019.00165

**Published:** 2019-03-29

**Authors:** Maxim B. Gongalsky, Julia V. Kargina, Jose F. Cruz, Juan F. Sánchez-Royo, Vladimir S. Chirvony, Liubov A. Osminkina, Michael J. Sailor

**Affiliations:** ^1^Faculty of Physics, Lomonosov Moscow State University, Moscow, Russia; ^2^Department of Chemistry and Biochemistry, University of California, San Diego, San Diego, CA, United States; ^3^Institute of Material Sciences, University of Valencia, Valencia, Spain; ^4^Institute for Biological Instrumentation of Russian Academy of Sciences, Moscow, Russia

**Keywords:** silicon nanoparticles (SiNPs), photoluminescence, biomedical application, theranostics, porous silicon (PS)

## Abstract

We propose a rapid, one-pot method to generate photoluminescent (PL) mesoporous silicon nanoparticles (PSiNPs). Typically, mesoporous silicon (meso-PSi) films, obtained by electrochemical etching of monocrystalline silicon substrates, do not display strong PL because the silicon nanocrystals (nc-Si) in the skeleton are generally too large to display quantum confinement effects. Here we describe an improved approach to form photoluminescent PSiNPs from meso-PSi by partial oxidation in aqueous sodium borate (borax) solutions. The borax solution acts to simultaneously oxidize the nc-Si surface and to partially dissolve the oxide product. This results in reduction of the size of the nc-Si core into the quantum confinement regime, and formation of an insulating silicon dioxide (SiO_2_) shell. The shell serves to passivate the surface of the silicon nanocrystals more effectively localizing excitons and increasing PL intensity. We show that the oxidation/dissolution process can be terminated by addition of excess citric acid, which changes the pH of the solution from alkaline to acidic. The process is monitored *in situ* by measurement of the steady-state PL spectrum from the PSiNPs. The measured PL intensity increases by 1.5- to 2-fold upon addition of citric acid, which we attribute to passivation of non-radiative recombination centers in the oxide shell. The measured PL quantum yield of the final product is up to 20%, the PL activation procedure takes <20 min, and the resulting material remains stable in aqueous dispersion for at least 1 day. The proposed phenomenological model explaining the process takes into account both pH changes in the solution and the potential increase in solubility of silicic acid due to interaction with sodium cations.

## Introduction

Porous silicon nanoparticles (PSiNPs) are promising agents for therapy and diagnostics (theranostics) of various diseases due to their biocompatibility (Canham, 1995; Durnev et al., 2010) and biodegradability (Canham, 2007). Therapeutic modalities include sensitizing of light (Osminkina et al., 2011) and ultrasound (Sviridov et al., 2017) resulting in oxidative stress, hyperthermia, or other cell/tissue damage mechanisms, in addition to targeted drug delivery (Park et al., 2009). The dissolution of the material can be tuned over a wide temporal range, allowing sustained release of drug over periods of several minutes to several months (Low et al., 2009; Park et al., 2009). Diagnostic modalities include fluorescent labeling (Gu et al., 2013) and contrast agents for magnetic resonance (Erogbogbo et al., 2012; Gongalsky et al., 2015). Fluorescence labeling with photoluminescent PSiNPs benefits from the near-IR emission of the material, which coincides with the infrared transparency window of human tissues, and from the long luminescence lifetime (in microsecond range), which allows suppression of tissue autofluorescence by time-gated imaging (Gu et al., 2013). The combination of its drug loading capabilities and its infrared photoluminescence properties presents interesting opportunities for theranostics (Kumeria et al., 2017).

Mesoporous silicon prepared by electrochemical etch of highly doped p-type Si typically requires an activation step in order to display strong photoluminescence (PL), because the silicon nanocrystals generated in the etch are too large to exhibit quantum confinement effect (Cullis et al., 1997). Reduction of the silicon core size is usually accomplished by oxidation of the PSi surface to generate silicon oxide phases (Park et al., 2009; Pavlikov et al., 2012; Joo et al., 2014). The process is accompanied by formation of an electronically insulating oxide shell, which improves confinement of charge carriers in the silicon nanocrystals and partially eliminates non-radiative recombination centers. As a result highly luminescent Si/SiO_2_ core/shell quantum dots are formed inside the porous matrix. The oxidation reaction retains the mesoporous morphology and thus allows subsequent drug loading (Park et al., 2009).

Activation of PL can be achieved by either liquid (Park et al., 2009; Joo et al., 2014) or gas phase oxidation chemistries (Pavlikov et al., 2012). A convenient and rapid means to induce oxidation is with a mildly basic aqueous solution of sodium borate (Joo et al., 2014). In that case, the optimal core size can be reached after between 50 and 400 min of oxidation, depending on borate and nanoparticle concentration; further dissolution results in loss of material and decrease in net PL signal. A drawback of the approach is that it is difficult to terminate the oxidation reaction in a reproducible manner. Usually the reaction is terminated by sudden dilution into excess deionized water, but this does not give consistent results. There is a tradeoff between speed of reaction and reproducibility: the faster the reaction, the more difficult it is to terminate the reaction at the point where quantum yield is maximum.

In this study we present a rapid, one-pot procedure to prepare photoluminescent PSiNPs that combines 2 stages: (i) oxidation in concentrated borax solutions, and (ii) instant termination of the reaction by addition of citric acid to rapidly change the pH to the acidic range where the rate of oxide dissolution is negligible.

## Materials and Methods

### Materials

Silicon wafers were obtained from Virginia Semiconductors Inc., sodium tetraborate decahydrate, citric acid, and rhodamine 6G were purchased from Sigma Aldrich Chemicals.

### Preparation of PSiNPs

Perforated mesoporous silicon films (Perf-PSi) were prepared by electrochemical etch of highly doped (specific resistivity −0.001 to 0.005 Ohm^*^cm) *p*-type Si wafers. A 3:1 by volume mixture of 48% aqueous HF and absolute ethanol was used. We used perforated etching, by application of a current density waveform consisting of 400 mA/cm^2^ for 0.36 s followed by 50 mA/cm^2^ for 1.8 s, repeated for 200 cycles (Qin et al., 2014). The resulting PSi films were then lifted off the substrate by etching in dilute HF solution (1:20 by volume mixture of aqueous 48% HF and absolute ethanol), for 5 min under an applied bias of 8 V. The freestanding films were then placed in deionized water and PSiNPs were obtained by subjecting the dispersion to ultrasonic fracture (50T, VWR international) for 24 h. Operating frequency was 35 KHz, ultrasonic power was 48 W in a 1.9 L tank, which corresponded to a power density of approximately 25 mW/cm^3^. The nanoparticles were then purified: the larger microparticles were removed by allowing the suspension to sit undisturbed for 24 h and the sediment (consisting of larger particles) was removed from the nanoparticle suspension and discarded. The smaller nanoparticles were then removed by subjecting the supernatant to 3 cycles of centrifugation (15 min, 15,000 rpm). For this stage of the process, the supernatant was discarded after each cycle, and the sedimented pellet of nanoparticles was re-suspended by brief ultrasonication. The resulting size-selected nanoparticles were then activated for PL by exposure to sodium tetraborate decahydrate (borax) Na_2_B_4_O_7_·10H_2_O solutions. The borax concentration was varied in the range from 0.2 to 16 mM in order to evaluate the dependence of borax concentration on the oxidation reaction rate.

### Characterization

PL of the suspensions was monitored *in situ* in the spectral range from 500 to 1,000 nm using an OceanOptics QE-65 spectrometer fitted with a 460 nm long-pass emission filter. The suspensions were placed in UV-transparent parallelepipedal cuvettes with length and width equal to 1 cm. The excitation source was a 365 nm light emitting diode (LSM-365A) with emission power about 10 mW and full width at half maximum (FWHM) about 12 nm. All elements of PL setup were coupled by optical fiber with SMA 905 connectors. The cuvettes were set into cuvette holder with 4 ports located on the 4 sides. Emission light beam was focused into the center of the cuvette via direct port. PL signal was collected by using of condenser also focused into the center of the cuvette, which was connected via side port of the holder. Typical exposure time was 3 s.

The quantum yield (QY) of PSiNPs was measured by the comparative method, using Rhodamine 6G as a standard (Kubin and Fletcher, 1982). In this approach integrated PL intensity/absorption ratio is compared for the investigated and reference samples. At least 3 different concentrations of Rhodamine 6G and PSiNPs were used to confirm linearity of the dependency. Optical absorbance was <0.2 at 365 nm for all QY measurements. Excitation and absorption wavelengths must coincide (365 nm in this study). QY of Rhodamine 6G was assumed to be 95%. PL was measured using the setup described above. Absorbance measurements were made using a SpectraMax 340PC384 reader from Molecular Devices in the same cuvettes as were used for PL measurements. Spectra were measured in the range from 300 to 850 nm, but only values at 365 nm were used in the analysis. Optical depth was measured in the range from 0 to 4 with 0.006 accuracy.

Dynamic light scattering (Malvern Instruments Zetasizer ZS90) was used to determine average size (hydrodynamic diameter) of PSiNPs. The setup uses a 633 nm, 4 mW laser backscattered from suspended nanoparticles in the same cuvette that was used for PL and absorbance measurements. Precise photon counting was used for continuous measurements of the scattering intensity, calculation of the auto-correlation function, and final hydrodynamic distribution weighted by volume of nanoparticles. The mathematical model was based on the Smoluchowski diffusion equation.

**Figure 1:**
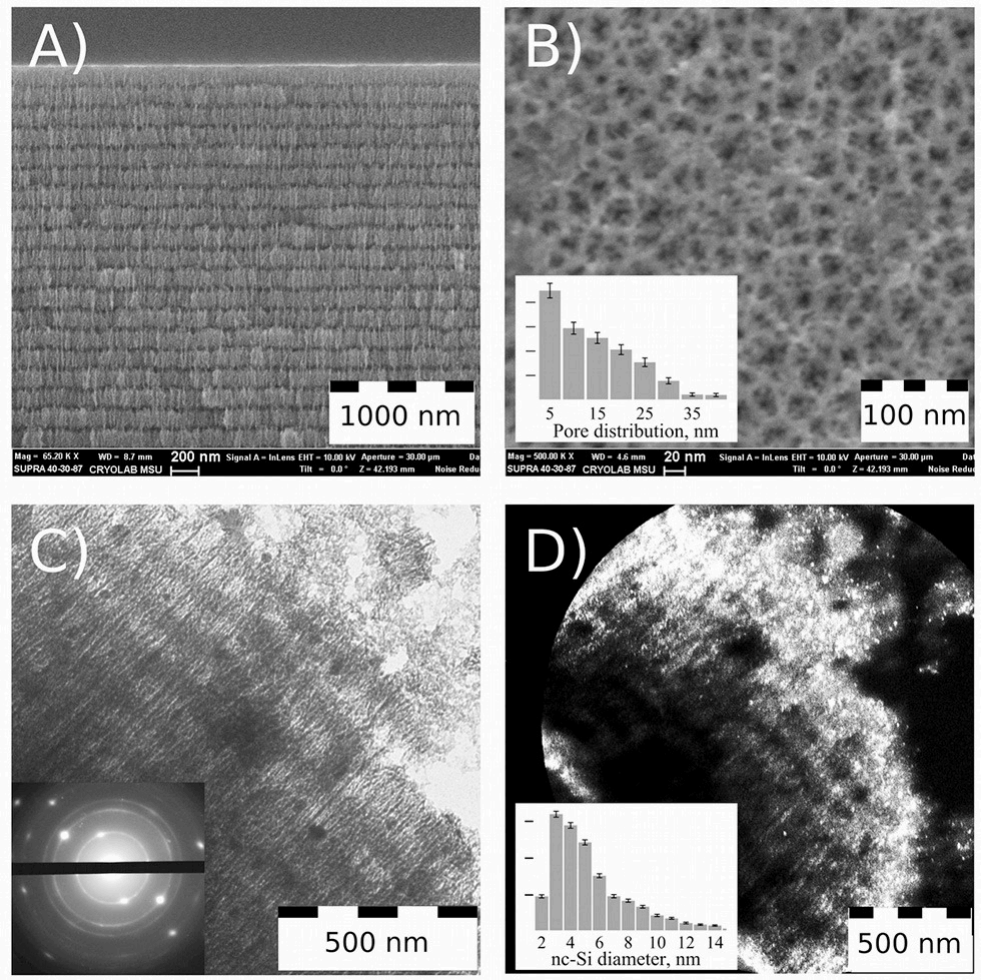
Electron microscope study of Perf-PSi. SEM images of Perf-PSi layer: cross-sectional view **(A)** and plan-view **(B)**; Pore distribution, calculated from SEM image (inset **B**); TEM image of Perf-PSi piece, that was isolated midway through the ultrasonic fracturing process **(C)**; Electron diffraction pattern corresponding to TEM image (inset **C**); Dark-field image of the same piece **(D)**. Bright spots correspond to silicon nanocrystals; Diameter distribution of the nanocrystals (inset **D**).

Structural investigations of the samples were carried out with a field emission scanning electron microscope (Carl Zeiss ULTRA 55 FE-SEM) operated at an acceleration voltage of 10 kV. Transmission and reflection Fourier-transform infrared (FTIR) spectra of PSi layers were measured with a Bruker IFS 66 v/S FTIR spectrometer. TEM images were obtained with a LEO 912 AB Omega transmission electron microscope (Zeiss, Oberkochen, Germany) operated at an acceleration voltage of 120 kV. Size distributions (in the insets of Figures 1B,D) were obtained from the electron microscope images using ImageJ software. Each image was modified by contrast enhancement in GIMP software and then dark/bright spots corresponding to pores/crystallites were outlined and fitted by ellipses. Each ellipse gave us two values, i.e., major and minor axes, which both were used for calculation of pore/crystallite diameter distribution.

Raman scattering spectrum were measured with a Horiba Jobin-Yvon HR800 spectrometer with an He-Ne cw laser (wavelength of 633 nm, 5 mW) for the excitation.

X-ray Photoelectron Spectroscopy (XPS) investigations of Si nanoparticles were performed in an ultrahigh vacuum system ESCALAB210 (base pressure 1.0 × 10^−10^ mbar) from Thermo VG Scientific. The nanoparticles in suspension were dripped onto a Cu substrate just before the XPS measurements. The measurements were taken over an area of 1 mm^2^ of the densely covered Cu substrate. Photoelectrons were excited by using the Mg K_α_ line (1253.6 eV).

The pore diameter distribution was determined by using N_2_ adsorption/desorption isoterms (Quantachrome NOVA 4200e, Quantachrome Instruments). The specific pore area was determined from the adsorption branch using BET theory (Brunauer et al., 1938), and the pore size distribution was calculated from the desorption branch using BJH theory (Barrett et al., 1951). Samples were subjected to degasation at 300°C before measurements.

Porosity of PSi films was measured by spectroscopic liquid infiltration method (SLIM), which is based on comparison of 2 optical interferograms for dry film and film with pores filled by methanol, which alternates effective medium refractive index (Segal et al., 2007).

## Results and Discussion

PSiNPs were prepared using the perforated-etching procedure (Qin et al., 2014). The perforated etch generated Perf-PSi films with a multilayered periodic structure consisting of alternating high and low porosity, as revealed by scanning electron microscope (SEM) images (Figure 1A). Thickness of the primary layers (those with the lower porosity—about 45%) was in the range of 150–200 nm, while the perforation layers were 50 nm thick and had a porosity of ~80%. Thickness of the primary layers determines the final size of the PSiNPs. A plan-view SEM image (Figure 1B) reveals the pore morphology of the top primary layer, which exhibits hierarchical branching from larger (15–40 nm) to smaller (5 nm or less) pores. The quantitative pore distribution is given in the inset of Figure 1B, which was obtained using ImageJ software (details in Figure S1). The majority of the pores exhibited sizes in the range of 2.5–15 nm. It should be noted that the spatial resolution of the SEM is such that pores smaller than 2 nm would not be observable.

TEM images were acquired from a piece of Perf-PSi that was isolated midway through the ultrasonic fracturing process, after sedimentation from aqueous suspension (Figure 1C). The sample displays the layered structure of the parent Perf-PSi material, and cracking of the highly porous “perforating” layer is evident on the right top corner of Figure 1C (Full TEM image is shown in Figure S2). The electron diffraction pattern (inset of Figure 1C) displays several bright, distinct diffraction spots corresponding to bulk Si, confirming the presence of crystalline silicon in the Perf-PSi sample. The diffraction spots are attributed to Si nanocrystals that inherited their preferred orientation from the initial c-Si wafer. The diffraction rings present in the image can be attributed to other randomly oriented Si nanocrystals.

Figure 1D shows a dark-field image of the same Perf-PSi piece, where bright spots correspond to diffraction from nanocrystalline silicon selected by special positioning of the TEM diaphragm. The brightest regions correspond to the periphery of the Perf-PSi piece. Quantitative analysis yielded the distribution of silicon nanocrystallite diameters (inset to Figure 1D, detail—also in Figure S3) According to the analysis, the mean diameter of silicon nanocrystallites was 5.5 nm ± 0.5 nm, but a substantial quantity of larger silicon nanocrystallites (7–14 nm) is also present.

**Figure 2:**
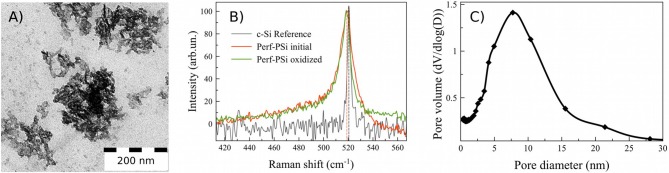
TEM image of PSiNPs, sedimented from aqueous suspension **(A)**; Raman spectra of initial Perf-PSi (red), oxidized Perf-PSi (green), and reference c-Si (black) **(B)**. Pore diameter distribution of PSiNPs sedimented from the aqueous suspensions by using the low-temperature adsorption technique **(C)**.

Digital photos of Perf-PSi layers and suspensions are shown in Figures S5A–C. Sequential sedimentation and centrifugation steps after ultrasonic fracturing gave the specified 200 nm sized PSiNPs; the average size and the size distribution of the resulting nanoparticles were confirmed by TEM and DLS measurements (Figure 2A and Figure S6). Figure 2A shows the network of pores was maintained after the ultrasonic fracture process. Each PSiNP can be thought of as an assembly of many smaller silicon nanocrystals (nc-Si). Measured Zeta potential of the PSiNPs was ~-30 mV (Figure S7), which imparted reasonable stability to the colloidal suspension.

The mean diameter of the silicon nanocrystallites was estimated from Raman and PL measurements from the peak position governed by the quantum confinement effects. Figure 2B shows the Raman spectra of the initial Perf-PSi film prior to oxidation (colored red), the Perf-PSi film after oxidation in aqueous borate solution (colored green) and bulk crystalline silicon (c-Si—black color) as a reference. The calculations (shown in Supplementary Material) give d_R_ = 5 nm ± 0.5 nm for the initial Perf-PSi film. Oxidation of the sample results in a slight shift of the band to lower wavenumbers, which is attributed to a further decrease in the mean diameter of the silicon nanocrystallites. More importantly, oxidation modifies the higher energy shoulder of the band, which lies beneath the shoulder of the initial Perf-PSi Raman band. This suggests that the oxidation process reduces the diameter of the larger (>7 nm) nanocrystallites.

Figure 2C shows the pore diameter distribution for a powder of initial PSiNPs deposited from the aqueous suspension. Corresponding low-temperature nitrogen adsorption/desorption isoterms (BET/BJH isoterms) are shown in Figure S8. The mean pore diameter was about 8 nm, which is in good agreement with data presented in the inset of Figure 1B. However, the pore distribution in Figure 2C revealed low volume of micropores and mesopores smaller than 4 nm. According to BET model the specific surface of PSiNPs was 480 ± 20 m^2^/g. Assuming spherical shape of nc-Si their mean diameter can be estimated from the value of surface area as 5.4 ± 0.5 nm, which is also in a good agreement with TEM and Raman results.

Neither the initial mesoporous Perf-PSi film nor the PSiNPs generated from ultrasonic fracture exhibited efficient PL. This is consistent with the microscopic and Raman data, which indicated that the average diameter of the majority of the silicon nanocrystallites in these samples were >5 nm (Figure 1D), too large to exhibit substantial quantum-confinement effects (Canham, 1990). In addition, the surface of the silicon nanocrystallites in these samples likely do not have a barrier silicon oxide layer sufficient to passivate non-radiative surface traps (Cullis et al., 1997).

**Figure 3:**
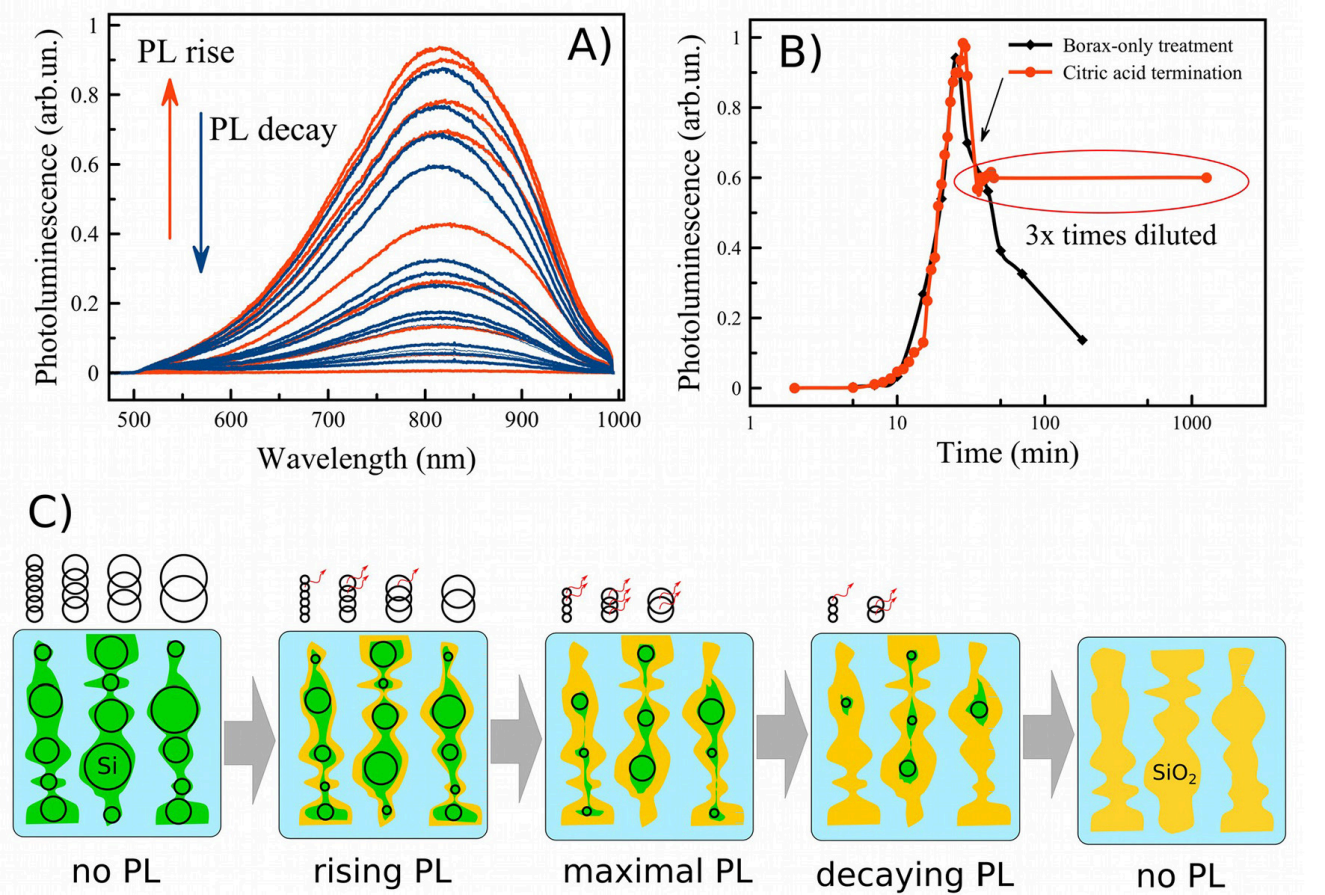
Family of PL spectra of porous silicon nanoparticles obtained during aqueous borax oxidation **(A)**; evolution of the PL intensity (measured at the emission maximum) during borax oxidation without (black curve—**B**) and with termination by citric acid (red curve—**B**); schematic view of the oxidation process starting from as-prepared PSiNPs through maximal emissive state to complete oxidation in borax **(C)**. Green—Si skeleton, yellow—SiO_2_ shell, black circles—silicon nanocrystals (nc-Si) indicating the size of the nanocrystals, some of which fit the quantum confinement criteria for efficient emission, indicated with the red arrows.

Two criteria are necessary to activate efficient PL: the average nanocrystallite size must be reduced into the quantum size regime, and the surface coating must be sufficiently passivated to inhibit non-radiative carrier recombination. In this work surface passivation was achieved with an SiO_2_ shell, which has been well-established to generate good surface passivation in PSiNPs. In this work we followed the aqueous borate oxidation/dissolution protocol previously reported (Joo et al., 2014). Figure 3A shows the time evolution of the PL spectrum from nanoparticles during treatment with an aqueous borax solution (concentration 6.5 mM). Initially, PL was not detected. Digital photo of luminescent suspension of PSiNPs under UV excitation is shown in Figure S5D. For the next 10–30 min in the borax solution, the PL spectrum appeared and grew in intensity. After a period of time the PL intensity reached a maximum, and then it decreased again (Figure 3B—black curve). The data are consistent with a model in which the borax solution induces both oxidation of the Si skeleton and dissolution of this oxide (Joo et al., 2014), simultaneously shrinking the nc-Si core and passivating the nc-Si surface, as outlined in Figure 3C: Starting from thick silicon nanowires (colored green) in borax solution (colored light blue) through to complete oxidation of the silicon nanocrystallites to non-emissive silicon oxide (colored yellow). The black circles on the picture represent the relative number of luminescent nanocrystallites present in the sample. In this model, PL intensity is approximately proportional to the number of emissive Si nanocrystals. As-prepared PSiNPs and nanoparticles at the early stages of oxidation may contain some silicon nanocrystallites that meet the quantum-confinement criteria (see Figure 1D), but they do not have sufficient barrier oxide layer (shown as yellow) to display efficient emission due to non-radiative exciton recombination (Cullis et al., 1997). Once the oxide layer is sufficiently passivating, the silicon nanocrystallites begin to emit PL. Small silicon nanocrystallites are known to have a smaller quantum yield, because of increasing singlet-triplet state splitting and increasing non-radiative recombination processes (Ledoux et al., 2002). Continuation of the oxidation process eventually leads to a decrease in the amount of silicon nanocrystallites present, and ultimately to complete oxidation of the sample, which is accompanied by the disappearance of PL.

The PL spectra presented in Figure 3A can be used to estimate the mean diameter of luminescent silicon nanocrystallites in the samples. The PL energy *E*_*PL*_ is roughly determined from the nc-Si diameter *d*_*PL*_ by:

(1) $$\begin{array}{l} E_{PL}=E_{g}+\frac{3.73}{d_{PL}^{1.39}}, \end{array}$$

where *E*_*g*_ is band gap of bulk Si (Ledoux et al., 2000). The peak position of 810 nm yields d_PL_ = 5 nm ± 0.5 nm, which is consistent with the values obtained from Raman and TEM data. The shift of the PL peak position is <20 nm, indicative of a relatively broad distribution of nc-Si sizes (Figure 1D). Thus, during the nc-Si oxidation process, the smaller end of the nanocrystal ensemble becomes fully oxidized and non-emissive, while the larger end of the nanocrystal ensemble becomes smaller and begins to contribute to the PL spectra. Ensembles with narrower size distributions of nanocrystallites might be expected to generate a more pronounced blue shift in the overall emission spectrum according to formula (2), as was seen in Joo et al. (2014).

Since efficient PL is desirable for many applications, it is important to capture the oxidation/dissolution process at the moment of maximal PL. On the one hand, borax oxidation is a rapid and convenient process, but on the other hand, it is difficult to terminate at the point where the PL emission is maximal. As is shown in Figure 3B, just a few minutes is sufficient to lose PL efficiency several-fold. Rapid dilution of the solution slows but does not stop the oxidation/dissolution process. We reasoned that acidification to change the pH of the solution from alkaline to acidic might be a more effective means to instantaneously terminate dissolution and freeze the overall process. When excess citric acid (CA) was used for this purpose, we found that pH changed from 9.3 to ~3 very rapidly, and PL was stabilized. The process is shown in the red trace of Figure 3B, and the moment of CA addition is indicated with an arrow. PL was stabilized within 1 min of CA addition. Under these conditions, PL intensity was stable for 1 day after CA addition.

**Figure 4:**
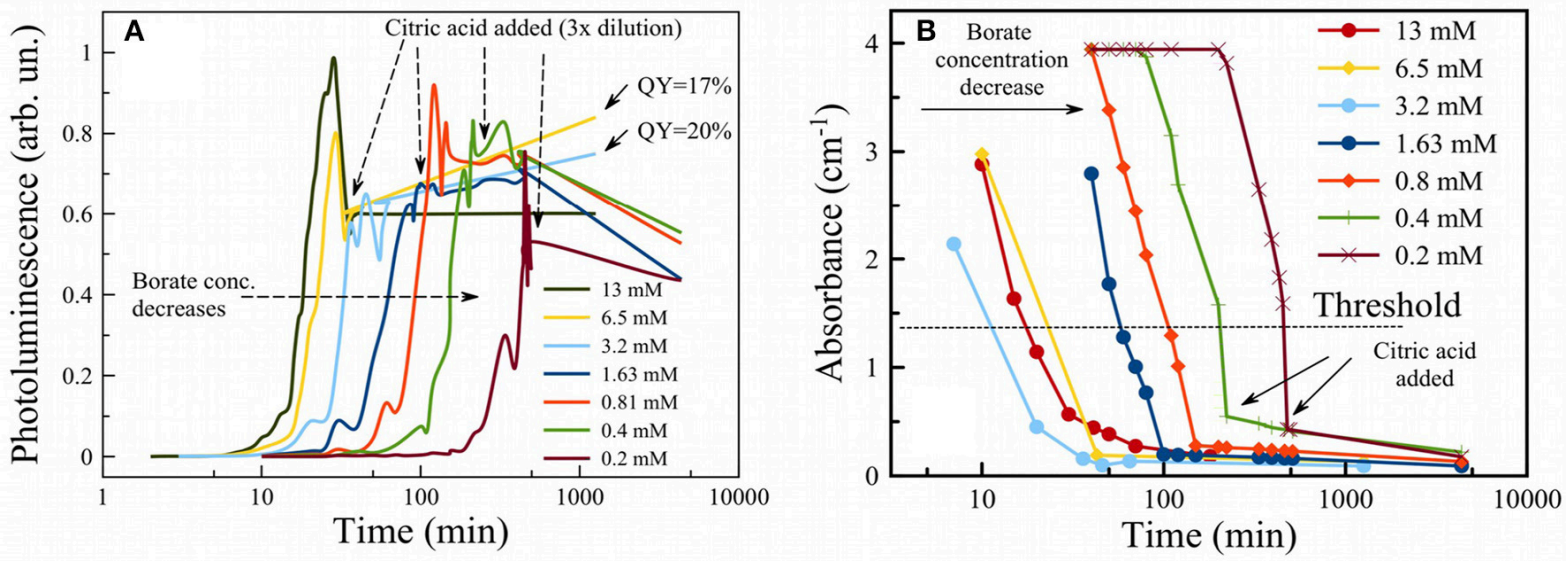
Photoluminescence of PSiNPs during oxidation in aqueous borax with addition of citric acid to terminate the reaction (showed by vertical arrows) **(A)**. Initial borate concentrations for each trace are given in the legend. Absorbance of PSiNPs during the same process **(B)**.

The citric acid solution was added to the PSiNPs/borax suspension in a 2:1 ratio by volume. PL intensity remained approximately the same, even though the concentration of PSiNPs dropped by 3-fold upon dilution. This suggests that CA treatment induced an overall increase in the PL QY of PSiNPs. The origin of this enhancement is unclear at this time, though it is possible that CA contributed to passivation of the surface and elimination of non-radiative recombination centers for excitons in nc-Si. QY of PSiNPs in such suspensions measured after 1 day of storage in CA ranged from 15 to 20% depending on borax concentration (see QY values pointed by arrows in Figure 4A).

The time needed to activate PL in borax solutions can be tuned by changing borax concentration (Figure 4A). One can see that the higher the concentration of borax, the faster the PL evolved to its maximum and then subsequently decayed. Activation times could be varied over a wide range: from 10 min to several hours. It was shown in previous work that 6 days are required to activate PL in deionized water (Park et al., 2009). The result obtained from the PL measurements was verified by measurement of optical absorbance of a PSiNP suspension also during oxidation in borax (Figure 4B). Absorbance measurements at 365 nm showed similar activation times as well as a drastic drop in absorbance after the point in time where PL was at a maximum. This also points to oxidation/dissolution of the Si skeleton, since neither silicon dioxide nor silicic acid absorb light of 365 nm wavelength. The data can thus be used to track the phase composition of partially oxidized PSiNPs. Although absorbance values for all samples were out of range in the beginning, useful data were obtained in the range 0.1 to 3 cm^−1^. The absorbance value corresponding to maximum PL was ~ 0.35 cm^−1^. Different concentrations of borax yielded different PL activation times, but the final QY after termination of the reaction with CA was similar and could be as high as 12%. That result differs from the observations in a previous study (Joo et al., 2014), where high concentrations of borax did not correspond to maximal QY. Therefore, we believe the use of CA provides a more reliable means to produce photoluminescent Si, although there is still the requirement to closely monitor the process *in situ*.

**Figure 5:**
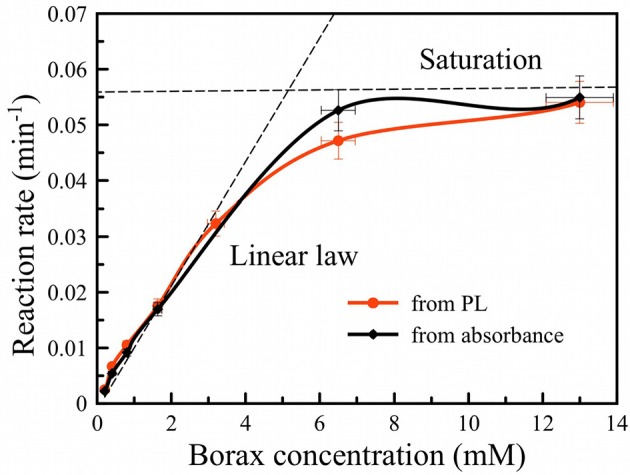
Oxidation rate of PSiNPs vs. borax concentration in suspension obtained from photoluminescence (red curve) and absorbance (black curve) measurements. Dashed line traces are included as a guide to the eye.

The reaction rate was estimated from the initial time dependences in Figures 4A,B assuming a single-exponential first order rate law and using the following data thresholds: for the PL evolution (Figure 4A), data from time = 0 to the time point at which PL intensity was half of its maximum value was used for the fit; for the Si absorbance data (Figure 4B), measured absorbance values between 3 and 0.5 were fit by linear function and the values of time corresponding to threshold shown as the dotted line (1.25) were used. The result is presented in Figure 5 for both PL (red curve) and absorbance (black curve). Both dependences appear similar, i.e., they show a linear dependence of rate on borax concentration at low values (increasing rate with increasing borax concentration) and saturation of the rate at high borax concentrations. The data are consistent with the strong pH dependence of silica dissolution. Silica is stable at low pH but it readily dissolves at high pH. In the borate-induced oxidation/dissolution process, borate acts as a buffer, maintaining the pH at ~9, where dissolution of the formed oxide can occur. At low concentration of borate, the buffer capacity is low and thus readily exceeded by the silica dissolution reaction, which can consume hydroxide ions to form [Si(O)(OH)_3_]^−^ ion (pKa = 10, Equation 3). As the pH drops, the dissolution of SiO_2_ will slow down, and because the SiO_2_ layer acts as a protective coating for the Si skeleton, the oxidation of silicon will also slow. Thus, increasing the borax concentration increases the rate of Si dissolution.

Saturation of the reaction rate can be explained by diffusion limitation of sodium tetraborate inside the pore network of PSiNPs. Borax depletes deep inside PSiNPs, thus the reaction slows down. Diffusion of new borax from the solution to the depth of PSiNPs is required for maintenance of the process, which is limited by diffusion through porous network. The diffusion coefficient of small molecules may vary in the wide range depending on pore diameter (Carbonaro et al., 2004). However, partial dissolution of PSiNPs may enlarge the pores and increase diffusion of reagents through the porous network.

**Figure 6:**
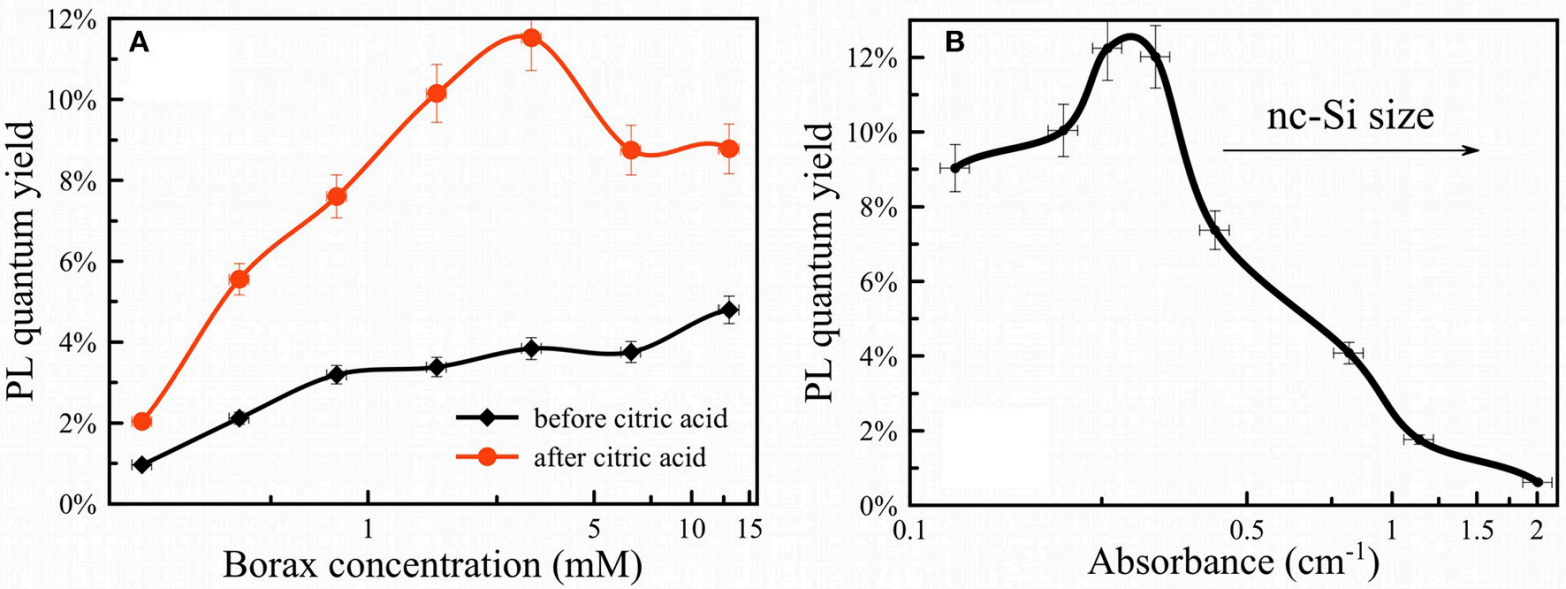
PL quantum yield of PSiNPs vs. borax concentration **(A)** before (black curve) and after CA termination (red curve); PL quantum yield of PSiNPs vs. their absorbance at 365 nm **(B)**. Higher absorbance corresponds to both a larger quantity of silicon and larger silicon nanocrystals.

The rate of Si dissolution was also measured indirectly by measurement of photoluminescence from the PSiNPs. PL QY showed a strong dependence on borax concentration (Figure 6A). An example of dependences of PL on absorbance used for calculation of QY is shown in Figure S9. High concentrations of borax (>1 mM) activated PL more quickly and provided the larger QYs (~10%). Low borax concentrations (<1 mM) led to loss of QY as well as a slowing of the oxidation process as described above. The optimal borax concentration from a perspective of experimental convenience and maximization of QY was between 2 and 20 mM.

The oxidation/dissolution process induced by borax could be interrupted by rapid acidification with citric acid (CA). Figure 6A shows the dependence of final measured QY after termination of the reaction with CA, presented as a function of borax concentration in the initial solution. The data are also presented as a function of optical absorbance of the sample in Figure 6B. The points from Figure 6B can be considered as the same sample subjected to the same oxidation process, but interrupted by CA addition at various times. As discussed above, absorbance correlated both with the overall amount of Si in the sample and the average diameter of nc-Si in the sample. Indeed, strong absorbance corresponds to larger nc-Si with a thin SiO_2_ shell and weak absorbance corresponds to smaller nc-Si inside an SiO_2_ shell. For these samples the larger nc-Si displayed a relatively low QY (below 1%). The QY rose if oxidation was allowed to proceed for a longer time. Surprisingly, QY did not drop substantially upon further oxidation (after PL intensity passed its maximum and began to degrade). This is interpreted to indicate that late stages of oxidation reduce the total amount of luminescent nc-Si, but the PL efficiency of individual silicon nanocrystallites is retained. Thus, the late stages of oxidation reduce the overall mass yield of PSiNPs, but not the PL QY. Furthermore, Figure 6A shows that addition of CA resulted in enhancement of the PL QY, by factors of 2–3 times for all the borax concentration conditions tested. This result suggests that CA plays an additional role in enhancing PL, possibly via passivation of surface defects rather than changing the nc-Si sizes. Note that all measurements in this phase of the experiments were performed when PL reached its maximum for a given borax concentration.

Based on the above results, we propose that the mechanism of PSiNP oxidation/dissolution follows the mechanism depicted in Figure 3C: after extensive borax treatment the nanostructure is converted completely into SiO_2_ without any Si core inside. Concomitant with the oxidation process is the dissolution of the SiO_2_ shell. This interpretation is consistent with prior reports; however the data reported here and below indicate that the process is slightly more complicated.

To further investigate the borax-induced oxidation/dissolution process, PSi films (shown in Figure 1B) were subjected to the same borax oxidation conditions and then analyzed by Fourier transform infrared (FTIR) spectroscopy.

**Figure 7:**
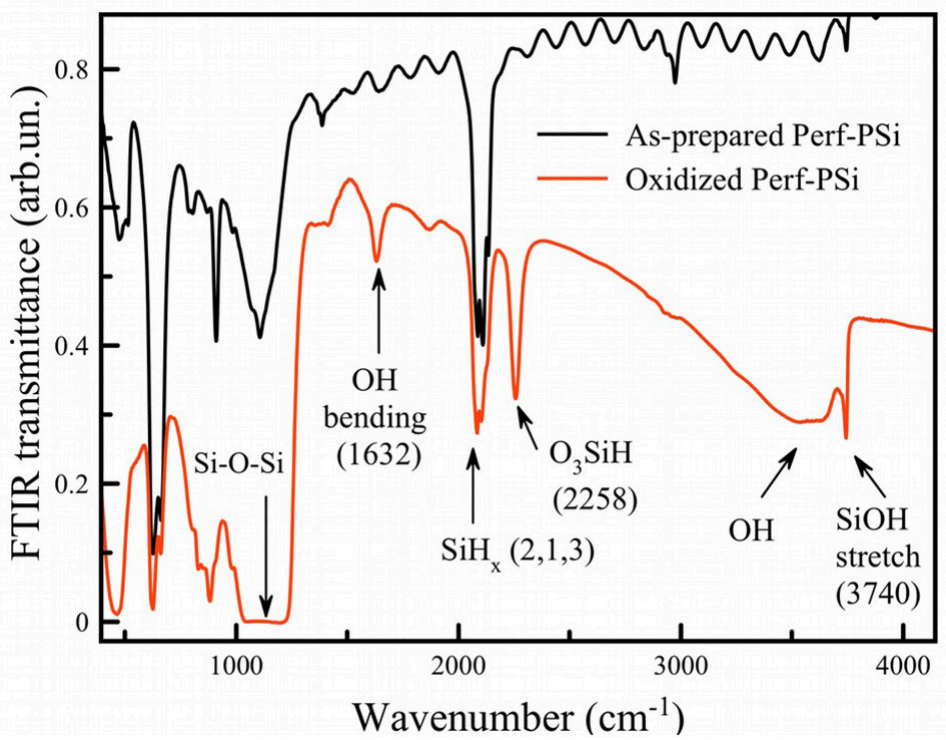
FTIR transmission spectra of as-prepared (black curve) and borax-oxidized (red curve) PSi films.

The spectrum of oxidized PSi (Figure 7; red curve) showed a strong absorption band at 1,070 cm^−1^ corresponding to stretching vibrations of Si-O-Si bonds in the SiO_2_ phase (Theiss, 1997). As-prepared PSi is characterized by strong Si-H stretching bands associated with surface Si-H, SiH_2_, and SiH_3_ species at 2,088 cm^−1^, 2,114 cm^−1^, and 2,137 cm^−1^, respectively (Gupta et al., 1991), along with other Si-H bands at 625 cm^−1^, 660 cm^−1^ (wagging Si_3_-Si-H, Si_2_-Si-H_2_), 910 cm^−1^ (scissors Si_2_-Si-H_2_) (Ogata, 1995; Theiss, 1997). As-prepared porous silicon is well-known to have a hydrogen-terminated surface. Surface hydrides were still detected after borax oxidation, although a new band associated with Si-H species that contain back-bonded Si-O was also present (2,258 cm^−1^, depicted as O_3_-Si-H in Figure 7). The data indicate that borate oxidation did not remove surface hydrides, or if it did, the silicon skeleton generated new surface hydrides via the chemical reduction of water.

Figure 7 exhibits a narrow band at ~3,740 cm^−1^ for oxidized Perf-PSi, which indicates the presence of isolated/free silanols (Morrow and Mcfarlan, 1992) as opposed to extensively hydrogen-bonded silanols (broad band centered at 3,500 cm^−1^). The intensity of the band was decreased after addition of CA, which may refer to the partial hydrolysis of the surface in acidic medium (Figure S10). Coexistence of both initial Si-H_x_ and oxidized O_3_-SiH groups points to a non-homogeneous oxidation process. Previous data show that oxidation takes place easier on convex regions, while smooth regions are less affected (Secret et al., 2015).

As discussed above, the mechanisms involved in PL activation need to take into account two simultaneous processes, i.e., oxidation of the Si core (nc-Si) and dissolution of the SiO_2_ shell. Oxidation can be induced by water or oxygen molecules (Ogata, 1995). Both molecules can attack Si-Si bonds forming Si-O-Si bonds as a result. The SiO_2_ shell is permeable to small molecules such as H_2_O and O_2_, because they can penetrate through small voids between irregularly positioned SiO_4_ tetrahedrons (Doremus, 1976). This process is strongly diffusion limited, therefore such an oxide layer is unlikely to be thicker than 1–2 nm to still allow molecular diffusion at room temperature.

Dissolution of silica in water is a very complex process characterized by numerous silicon-containing species and a strong pH dependence. A simplified representation of the process can be given with the following chemical reaction (Rimstidt and Barnes, 1980):

(2) $$\begin{array}{l} SiO_{2}\left(s\right)+2H_{2}O\left(l\right)=Si{\left(OH\right)}_{4}\left(aq\right) \end{array}$$

where (s), (l) and (aq) mean “solid,”, “liquid,” and “aqueous,” respectively. The process is reversible, so precipitation of solid silica from oversaturated silicic acid solution is possible. Equilibrium constants are different for different polymorphs, i.e., quartz, α-cristobalite, β-cristabalite, amorphous silica, etc. For example, the solubility of quartz is 35 times lower than amorphous silica (Walther and Helgeson, 1977; Rimstidt and Barnes, 1980). The kinetics of the reaction depends on the exposed specific surface area of the nanomaterial, which for the investigated samples was about 5·10^5^ m^2^/kg.

A significant increase (up to 50 times) in the solubility of SiO_2_ was observed for pH > 9 (Alexander et al., 1954). This is explained by deprotonation of the Si(OH)_4_ (silicic acid) product of Equation 3:

(3) $$\begin{array}{l} Si{\left(OH\right)}_{4}+{\left(OH\right)}^{-}=Si\left(O\right){\left(OH\right)}_{3}^{-}+H_{2}O \end{array}$$

The solubility of silicic acid is also affected by the chemical composition of the solution, not only by the value of pH (Piryutko, 1959). Borax increases the solubility of silica in proportion to its concentration in solution (Seward, 1974). For high borax concentrations there is 5- to 10-fold enhancement. We believe that factor thus accelerates PL activation, which is supported by the observed dependence on borax concentration shown in Figure 5.

The mechanism of the solubility enhancement has been proposed (Seward, 1974) to involve formation of ion pairs between sodium ions and the monovalent orthosilicate ion formed in Equation 4.

(4) $$\begin{array}{l} Na^{+}+Si\left(O\right){\left(OH\right)}_{3}^{-}=NaSi\left(O\right){\left(OH\right)}_{3} \end{array}$$

The reaction of Equation 5 is reversible and the system eventually reaches equilibrium, when concentrations of the various silicic acid species saturate. In porous structures like PSiNPs the saturation point is important, because local saturation can be achieved very quickly due to the high surface area of the material. Thus, the oxidation of silicon and the dissolution of silica in aqueous media depends on a number of factors, in particular the concentration of so-called “spectator” ions like sodium, the concentration of all the silicon-containing species, and the pH of the solution.

(5) $$\begin{array}{l} Na_{2}B_{4}O_{7}+SiO_{2}+H_{2}O+OH^{-}=NaSi\left(O\right){\left(OH\right)}_{3}+NaB_{4}O_{7}^{-} \end{array}$$

The state of the surface oxide may be a crucial determinant of the oxidation/dissolution rate. A thick oxide layer can protect against borax-induced corrosion of PSi. For instance, we verified that PSiNPs obtained by high energy mechanical milling and aged in aqueous solution for about 1 year were not affected by borax oxidation even in highly concentrated borax solutions. A similar effect has been observed for PSiNPs subjected to prior oxidation in H_2_O_2_ solutions. However, complete removal of the native oxide layer by rinsing in diluted HF solutions restored the ability of PSiNPs to be efficiently oxidized by the aqueous borax solution. This is likely related to the polymorphism of SiO_2_: for example, crystalline SiO_2_ is 10–100 times less soluble than amorphous silica or silicate glass (Walther and Helgeson, 1977).

The presence of defects in the silica shell nanostructure likely also influences solubility of PSiNPs. Dissolution of silicon dioxide (SiO_2_) has been described in terms of the two processes of hydration and hydrolysis; that is, diffusion of molecular water into the oxide and hydrolysis of surface species (Bunker, 1994). In general silica is a network of interconnected SiO_4_ tetrahedrons, and in the perfect situation each tetrahedron is connected to 4 others creating regular lattice such as in quartz. In quartz all oxygen atoms are connected with two silicon atoms, therefore they are called bridging oxygen. In amorphous silica there can also exist non-bridging oxygen groups, that is, oxygen atoms connected to only one silicon atom and having a charge of −1, and divalent oxide ions, i.e., oxygen atoms without any covalent connection to the SiO_2_ network with a charge of −2. Such non-bridging oxygen centers can exert substantial influence in the dissolution process (Bunker, 1994; Nesbitt et al., 2011).

**Figure 8:**
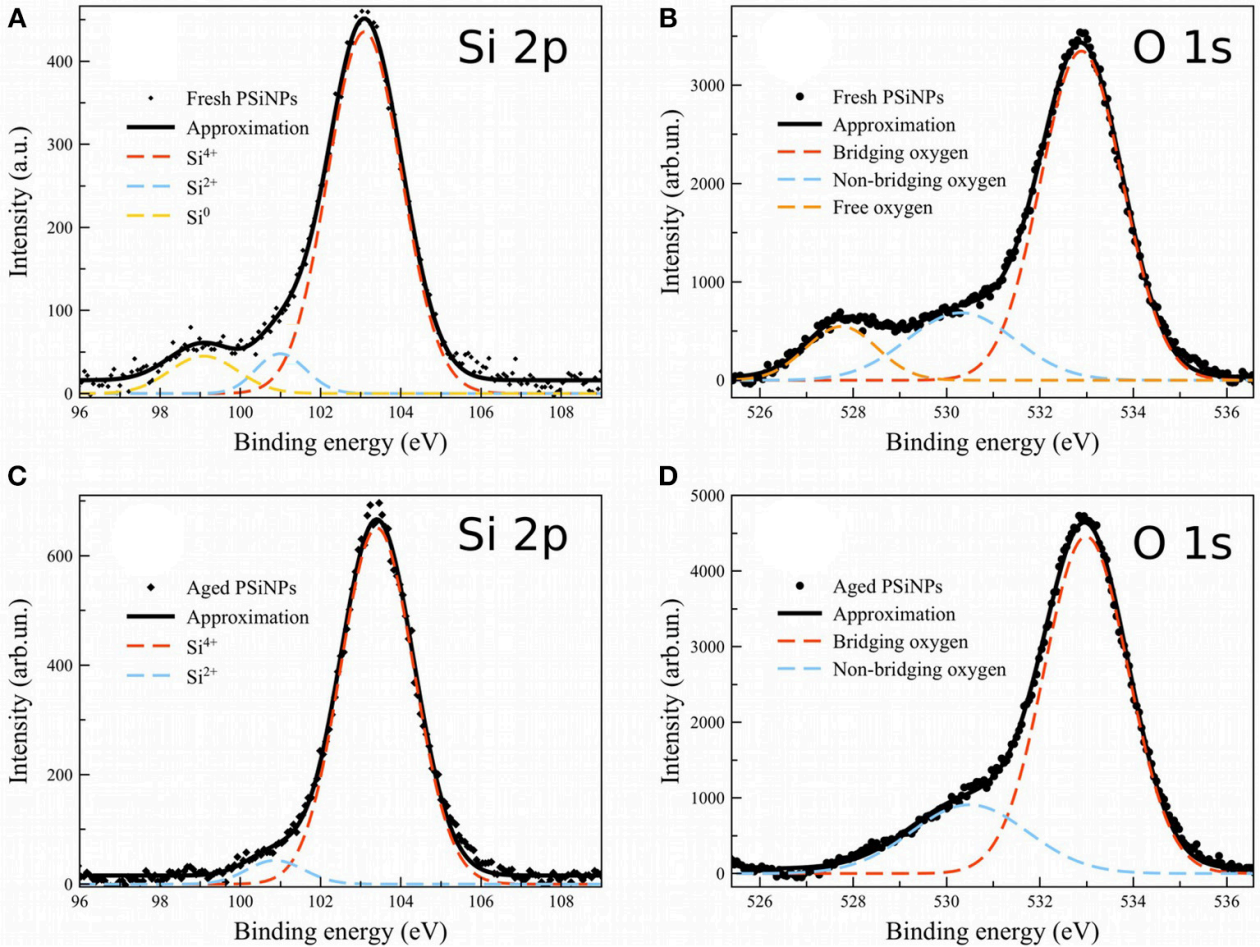
XPS spectra of “fresh” and “aged” PSiNPs. Dots: experimental data for “fresh” **(A,B)** and “aged” **(C,D)** PSiNPS; Solid lines: approximation; Dashed: deconvolution into Gaussians.

In order to gain more insight into the nature of the oxide shell in these materials, we acquired XPS spectra on both “fresh” and “aged” PSiNPs (Figure 8). The “fresh” PSiNPs were prepared as described above, that is perforated etching with subsequent ultrasonic fracture without any borax oxidation and the “aged” PSiNPs were prepared as described above (prepared from high energy mechanical milling and aged in aqueous solution for 1 year with concentration of approximately 20 mg/ml). Gaussian deconvolution of the Si 2p spectral region of fresh PSiNPs indicated the presence of three bands corresponding to completely oxidized silicon (Si^4+^–SiO_2_, ~103 eV), partially oxidized silicon (Si^2+^–SiO, ~101 eV) and unoxidized silicon (Si^0^–nc-Si, 99 eV). For aged PSiNPs, the latter band assigned to zerovalent silicon was absent (Grunthaner et al., 1979). This points to stronger oxidation of the “aged” PSiNPs, a thicker oxide shell and/or a stoichiometry close to SiO_2_. The O 1s spectra showed an abundance of free oxygen dianions (O^2−^, ~528 eV) in the “fresh” PSiNPs and an absence of free oxygen dianions in the “aged” PSiNPs. At the same, time both non-bridging (O^1−^, ~530 eV) and bridging (O^0^, ~533 eV) oxygen centers were detected in both samples (Nesbitt et al., 2011). The presence of free oxygen anions and the abundance of elemental, zerovalent silicon point to a non-homogeneous, disordered oxide shell in the “fresh” PSiNPs by comparison with the “aged” ones, which is consistent with the dissolution behavior and the differences in photoluminescence intensity observed. The oxide shell of the “aged” PSiNPs was more ordered and dense, containing fewer sites that might be subject to accelerated hydrolysis, consistent with the low susceptibility of this material to the aqueous borax solution. As mentioned above, removal of the protective layer by rinsing in aqueous HF completely recovered the ability of the “aged” material to be activated by borax oxidation.

**Figure 9:**
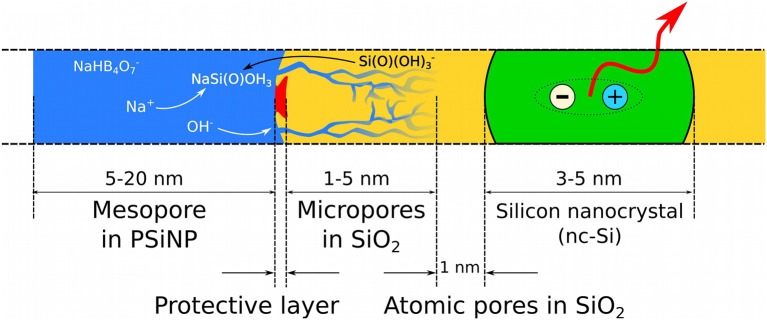
Schematic cross-section of a PSiNP subjected to borax-induced oxidation/dissolution. Borax solution (blue) penetrates into the SiO_2_ shell (yellow), which covers the silicon core silicon nanocrystallite (green). An exciton confined in the silicon nanocrystallite is shown as ≪ + ≫ and ≪ – ≫ in circles and photoluminescence is indicated with the red arrow. A notional insoluble protective surface layer is shown as a red region.

As a summary we present a phenomenological model for borax-induced oxidation/dissolution of porous Si based on the experimental data reported above. Figure 9 depicts the morphology of PSiNPs subjected to aqueous borax oxidation/dissolution. The scheme represents a cross-section of a silicon nanocrystallite (colored green) with an SiO_2_ shell (colored yellow) submerged in borax solution (colored blue), and with some of the mesopores in the PSiNPs infiltrated with the aqueous solution. Approximate sizes of regions are also given in the scheme. There is a diffusion limitation to the process, because locally high concentrations of silicic acid can slow the dissolution of SiO_2_ in the vicinity of the saturation. Creation of ion pairs between Na^+^ and Si(O)${\left(\text{OH}\right)}_{3}^{-}$ accelerates the dissolution process. Diffusion of OH^−^ into the micropores is also important, because it drives the dissolution of silica. Depletion of OH^−^ ions inside the micropores leads to a local pH drop, which slows the overall process. A protective insoluble layer (not always present) is depicted as the red region on the surface of the SiO_2_ shell. Notionally, this could be adsorbed polymer, protein, or some other species. In this model, if a protective layer covers the entire surface of PSiNPs, it will stop the oxidation/dissolution process completely.

The chemical oxidation of Si takes place at the Si/SiO_2_ interface (black line between the yellow and green regions in the diagram), which is known to be a typical location for point defects, such as dangling bonds, responsible for non-radiative recombination of quantum-confined excitons (shown as “+” and “–” in the scheme) (von Bardeleben et al., 1993). Reaction between the Si core and H_2_O or O_2_ leads to migration of the interface toward the center of the Si core, growing the SiO_2_ shell and shrinking the Si core. In the absence of defects an “atomic pore” layer characterized by highly restricted molecular diffusion partially protects the excitons from interaction with charged ions in the liquid phase, which determines the high quantum yield of PSiNPs. We propose that addition of citric acid terminates the growth of these micropores, allowing a thicker protective layer to grow that can increase quantum yield of the PSiNP.

## Conclusions

A new, relatively rapid and one-pot method was proposed for formation of highly luminescent porous silicon nanoparticles. The procedure can be accomplished in <20 min, and it generates Si-SiO_2_ core-shell nanoparticles with quantum yields up to 20% that remain stable in aqueous suspensions for at least 1 day. The time required for photoluminescence activation varies in a wide range depending on concentration of borax. Rapid addition of citric acid was found not only to instantly terminate the oxidation/dissolution of silicon, but it also increased quantum yield 2- to 3-fold depending on borax concentration. This was explained as the efficient passivation of non-radiative recombination centers by the acidic medium. Dependence of the reaction rate on borax concentration was linear at low values, but the rate tended to saturate at high values.

We propose that oxidation takes place via formation of micropores in the silica shell, which provides a supply of reagent to the silicon core, rather than uniform layer-by-layer dissolution. The constraints of diffusion inside narrow micropores may slow the overall rate of oxidation/dissolution. Borax plays a dual role in that it (i) sets up an alkaline medium with pH ~9 that accelerates dissolution of silica, and (ii) increases solubility of silicic acid. The latter effect is attributed to formation of ion pairs that prevents saturation of silicate ions in the pores. The reaction rate becomes constant in highly concentrated borax solutions, due to the pH buffering effect and mass transport limitations associated with removal of silicic acid from the spatially confined micropores.

## Data Availability

All datasets generated for this study are included in the manuscript and/or the Supplementary Material.

## Author Contributions

MG performed the majority of the experiments, analysis and wrote the manuscript. JK and JC assisted in PL measurements and sample characterization. JS-R and VC performed XPS experiments. LO and MS provided guidance for the research and editing of the manuscript.

### Conflict of Interest Statement

The authors declare that the research was conducted in the absence of any commercial or financial relationships that could be construed as a potential conflict of interest.
