# Supplementary figures and images for: Formation of Si/SiO2 Luminescent Quantum Dots From Mesoporous Silicon by Sodium Tetraborate/Citric Acid Oxidation Treatment
Source: Front Chem. 2019 Mar 29;7:165. doi: 10.3389/fchem.2019.00165 (PMC6450366; doi:10.3389/fchem.2019.00165)

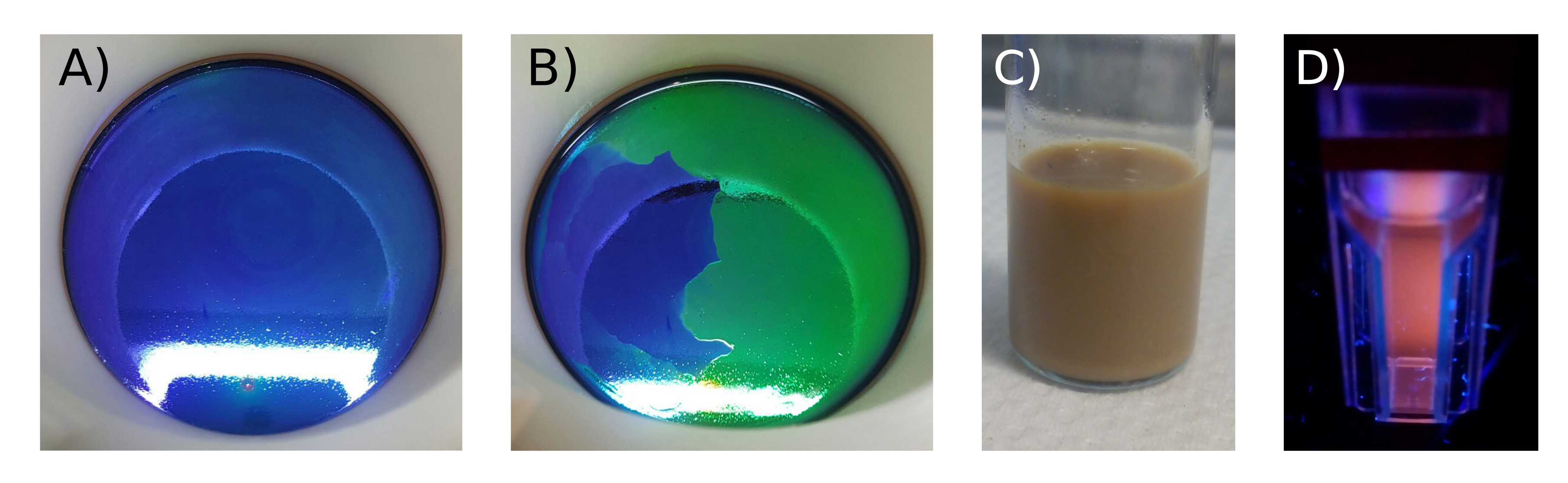

Supplement: Supplementary file 5 [file Image_5.TIFF]

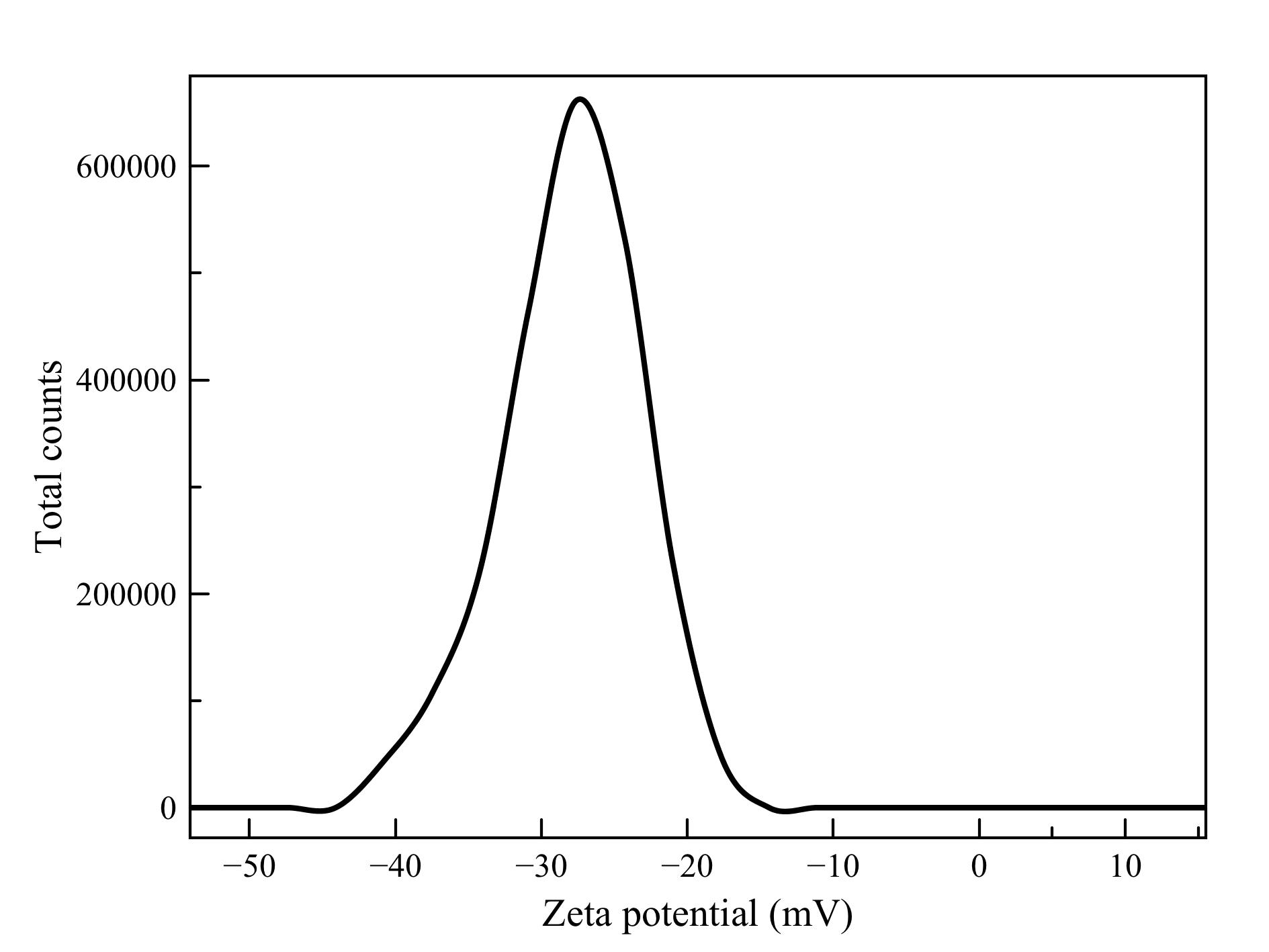

Supplement: Supplementary file 7 [file Image_7.TIFF]
