# Supplementary material for: Formation of Si/SiO2 Luminescent Quantum Dots From Mesoporous Silicon by Sodium Tetraborate/Citric Acid Oxidation Treatment
Source: Front Chem. 2019 Mar 29;7:165. doi: 10.3389/fchem.2019.00165 (PMC6450366; doi:10.3389/fchem.2019.00165)
Supplement: Supplementary file 11 [file Table_1.DOCX]

Supplementary material

Formation of Si/SiO_2_ luminescent quantum dots from mesoporous silicon by sodium tetraborate/citric acid oxidation treatment

M.B. Gongalsky^*^, J.V. Kargina, J.F. Cruz, J.F. SANCHEZ-ROYO, V.S. CHIRVONY, L.A. Osminkina, M.J. Sailor

**Corresponding author e-mail address: mgongalsky@gmail.com*


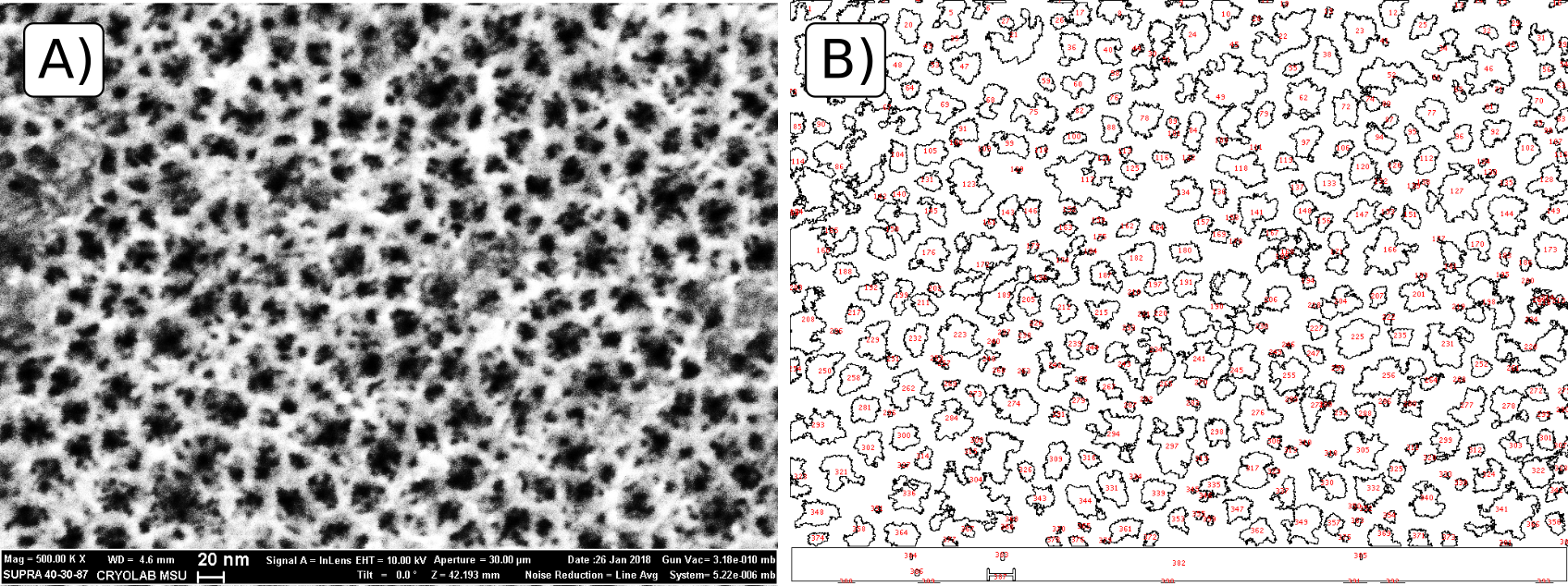


**FIGURE S1** | Enhanced contrast plan-view SEM image used for calculation of the size distribution of pores in Perf-PSi (A). Outlines of the pores used for calculation of the pore size distribution (B). Each outline was fitted by an ellipse and both minor and major axes of the ellipse were used. Each outline has a number inside.


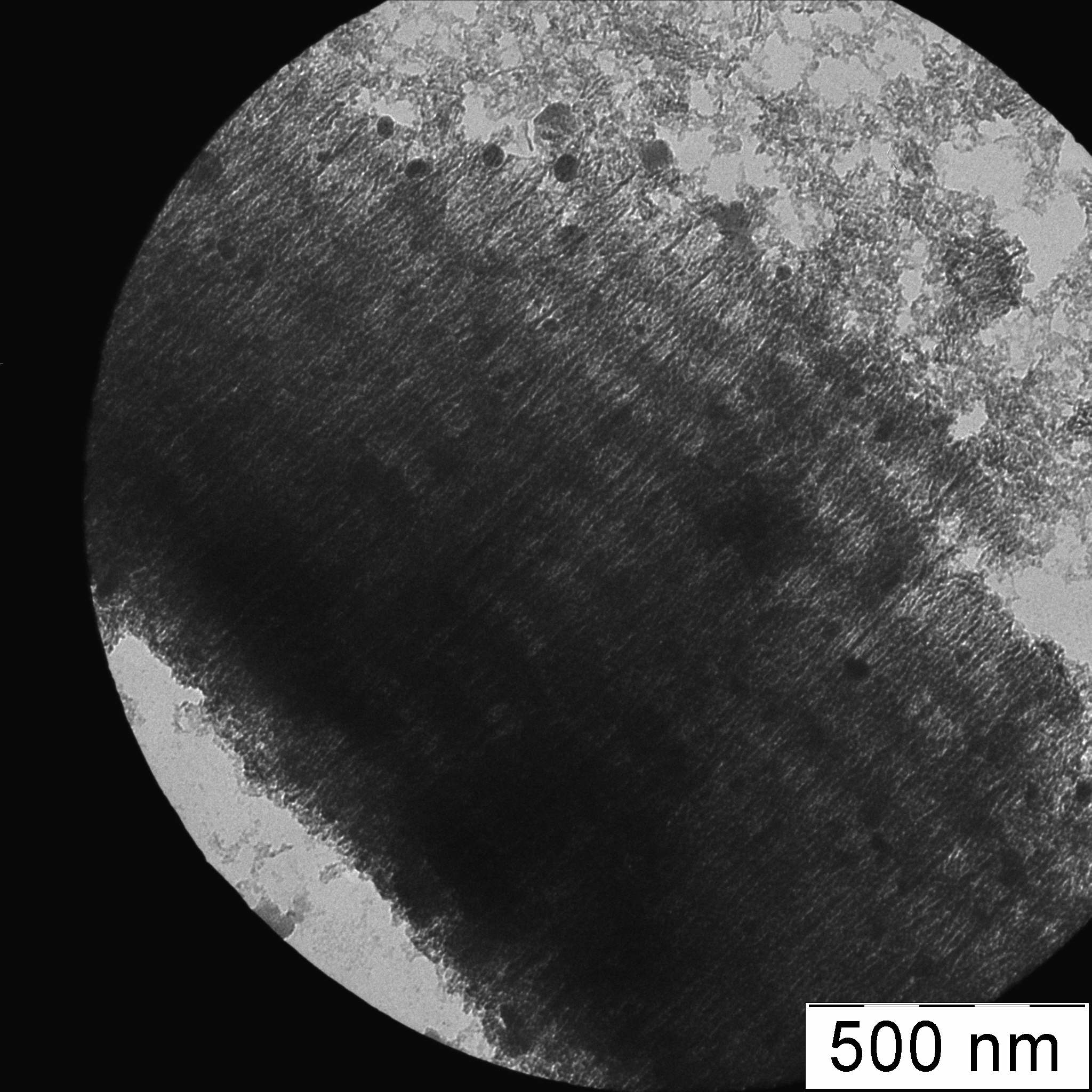


**FIGURE S2** | TEM image of Perf-PSi piece, that was isolated midway through the ultrasonic fracturing process (full view of 1C).


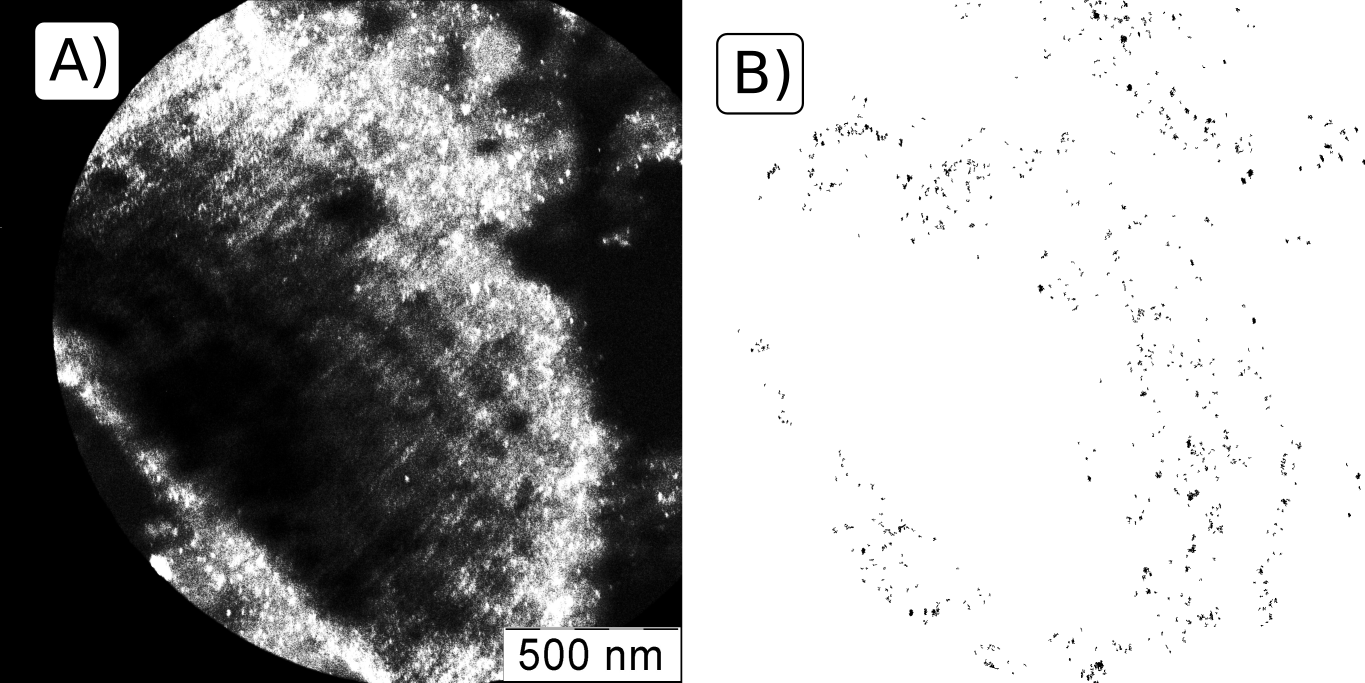
**FIGURE S3** | Enhanced contrast dark-field TEM image used for calculation of the crystallites distribution in Perf-PSi (A). Negative mask of the crystallites used for calculation of their distribution (B). Each black spot of the mask was fitted by an ellipse and both minor and major axes of the ellipse were used. Note, that mask includes only individual crystallites excluding regions with abundance of the intersecting crystallites, where calculation of their sizes is hindered.


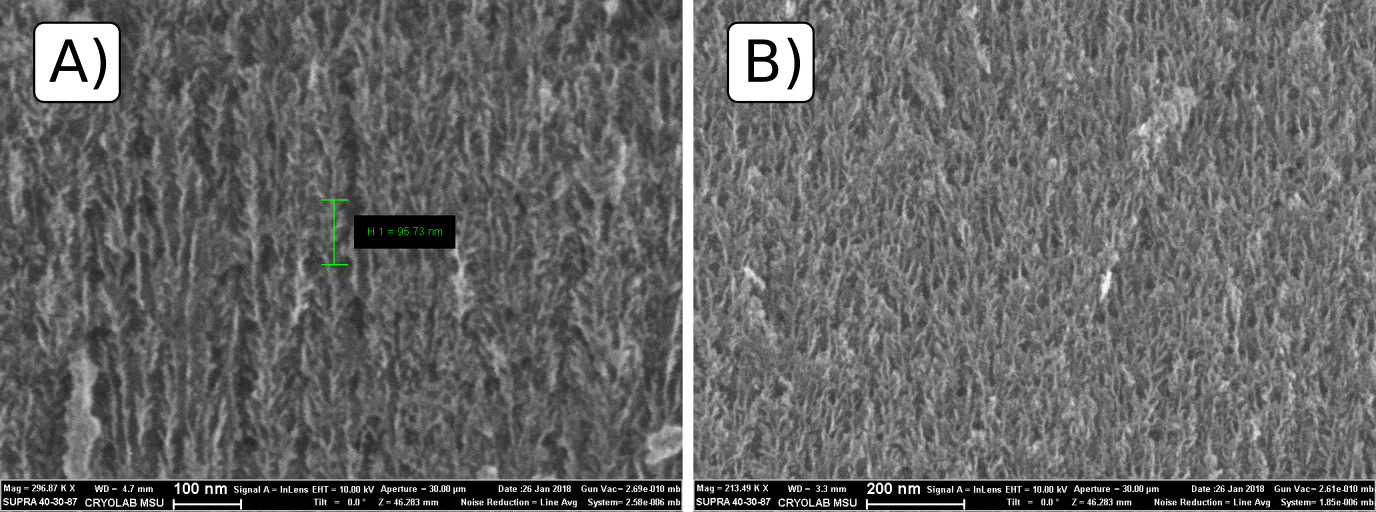
**FIGURE S4** | SEM images of Perf-Si layer. Cross-sectional view at the periphery of the layer: 0.5 cm from the edge (A), 0.2 cm from the edge (B). Perforation layers are less visible, period decreases below 100 nm (A). There is no periodic structure in (B).

**Description of visual properties of Perf-PSi layers and suspensions**

The alternating porosity in the layers in the Perf-PSi sample generates a dielectric stack, or photonic crystal, that produced a strong, relatively monochromatic reflection associated with the stop band of the photonic crystal (**Figure S5A**). The color of the sample depended on the relative layer thicknesses and their specific refractive index. The uniformity of the color gives a visual measure of the sample homogeneity, which was good even for 4-inch diameter samples. SEM microphotographs (**Figure 1A** and **Figure S2**) revealed the periodic structure, which became thinner and in some cases not observable at the periphery of the wafer. As expected for these types of porous nanostructures (Anderson et al., 2003)⁠, infiltration of ethanol changed the color of the structure by increasing the average refractive index of the layers (**Figure S5B**). This color change is an indication of the accessibility of the pores to small molecules. To prepare nanoparticles from these layers, the films were lifted off from the substrates and subjected to ultrasonic fracture in water to obtain aqueous suspension as shown in **Figure S5C**. When the resulting suspension was subjected to aqueous borate oxidation, detectable photoluminescence (PL) in the red and near-infrared region of the spectrum was observable under UV-excitation (**Figure S5D**).


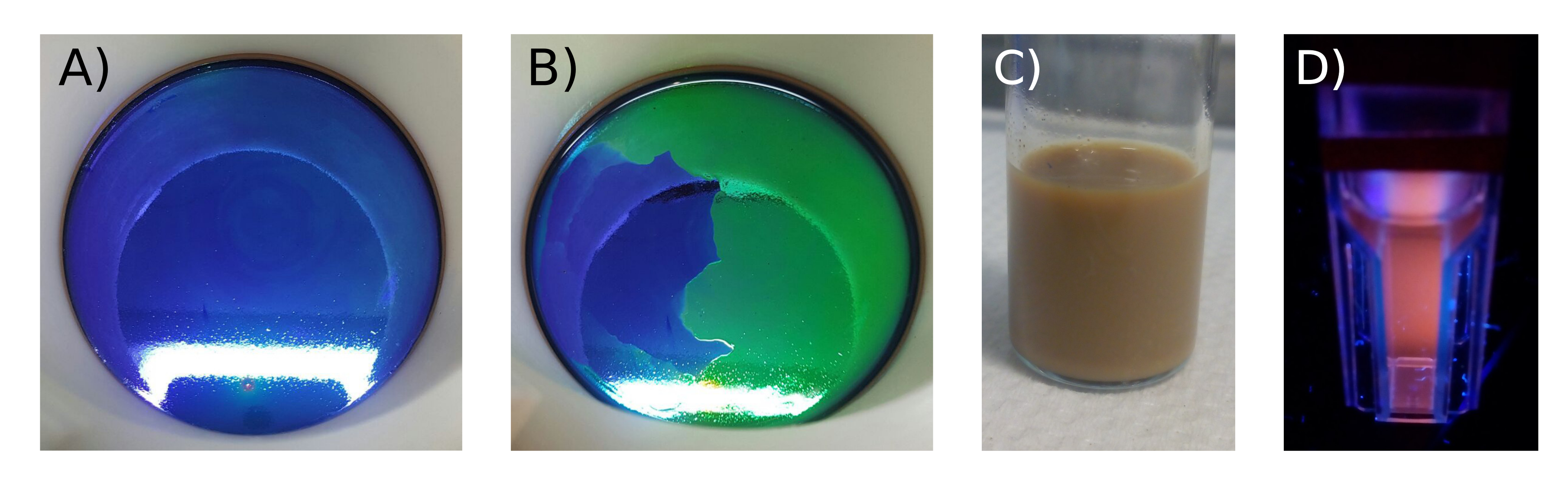


**FIGURE S5** | Digital photo of 4-inch Si wafer with dry Perf-PSi layer (A) and ethanol stain (green) (B) ; aqueous suspension of PSiNPs (C) ; Photoluminescence of borate-activated PSiNPs under UV-irradiation (D).

**Nc-Si mean diameter calculation from Raman spectra**

The Raman spectrum of c-Si is characterized by a narrow band at 520 cm^-1^ corresponding to scattering of transverse optical phonons in silicon (Richter et al., 1981)⁠. Porous silicon samples generally possess a similar band, but it is shifted and broadened due to phonon confinement effects in the nanocrystallites (Meier et al., 2006)⁠. The mean nc-Si diameter, d_R_, was estimated from the Raman spectra using the following formula (Paillard et al., 1999)⁠:

| , | (1) |
| --- | --- |

where Δω is the shift of the band relative to bulk crystalline silicon due to the phonon confinement.


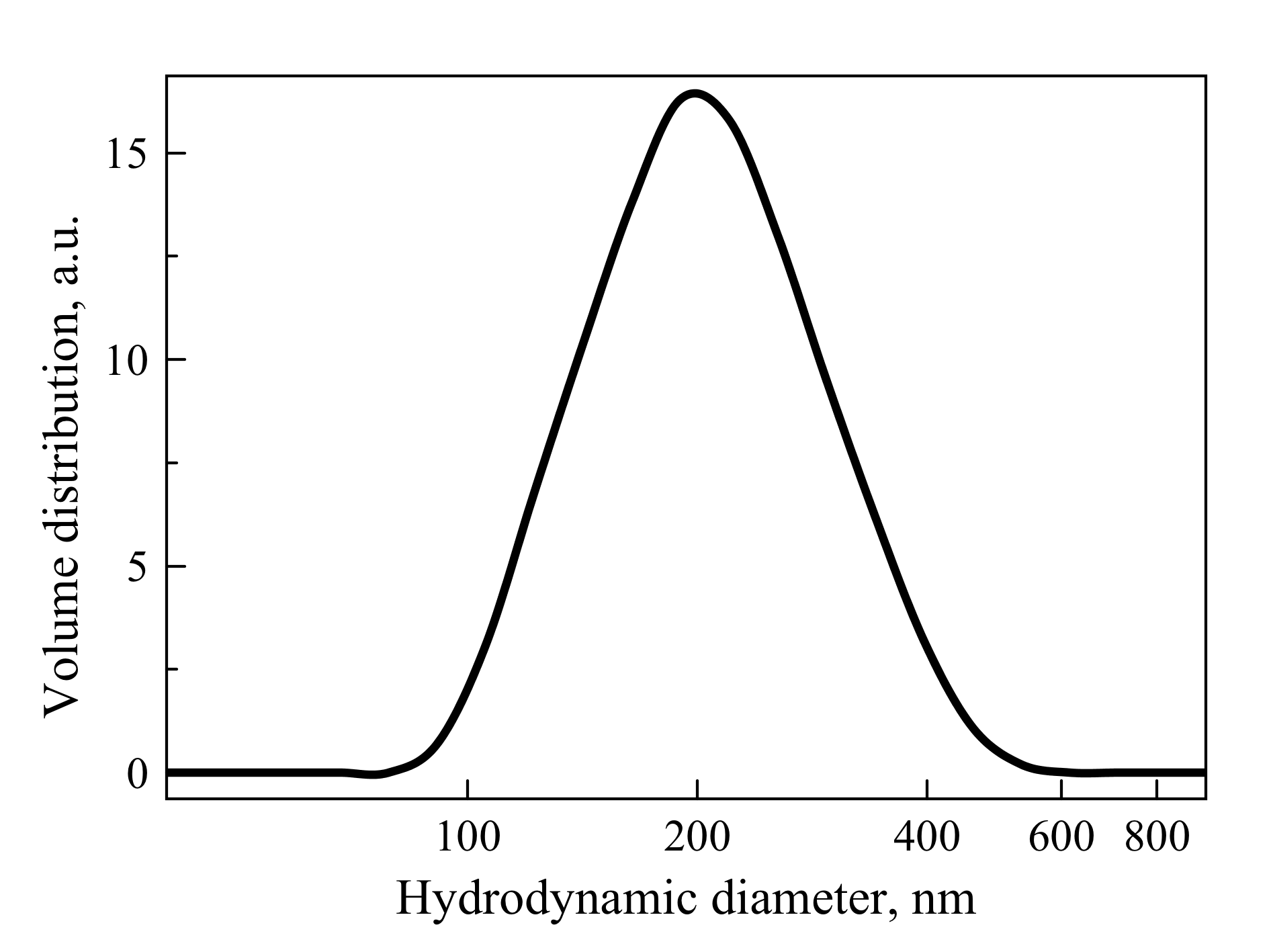


**FIGURE S6** | Hydrodynamic size distribution of PSiNPs in suspension by volume.


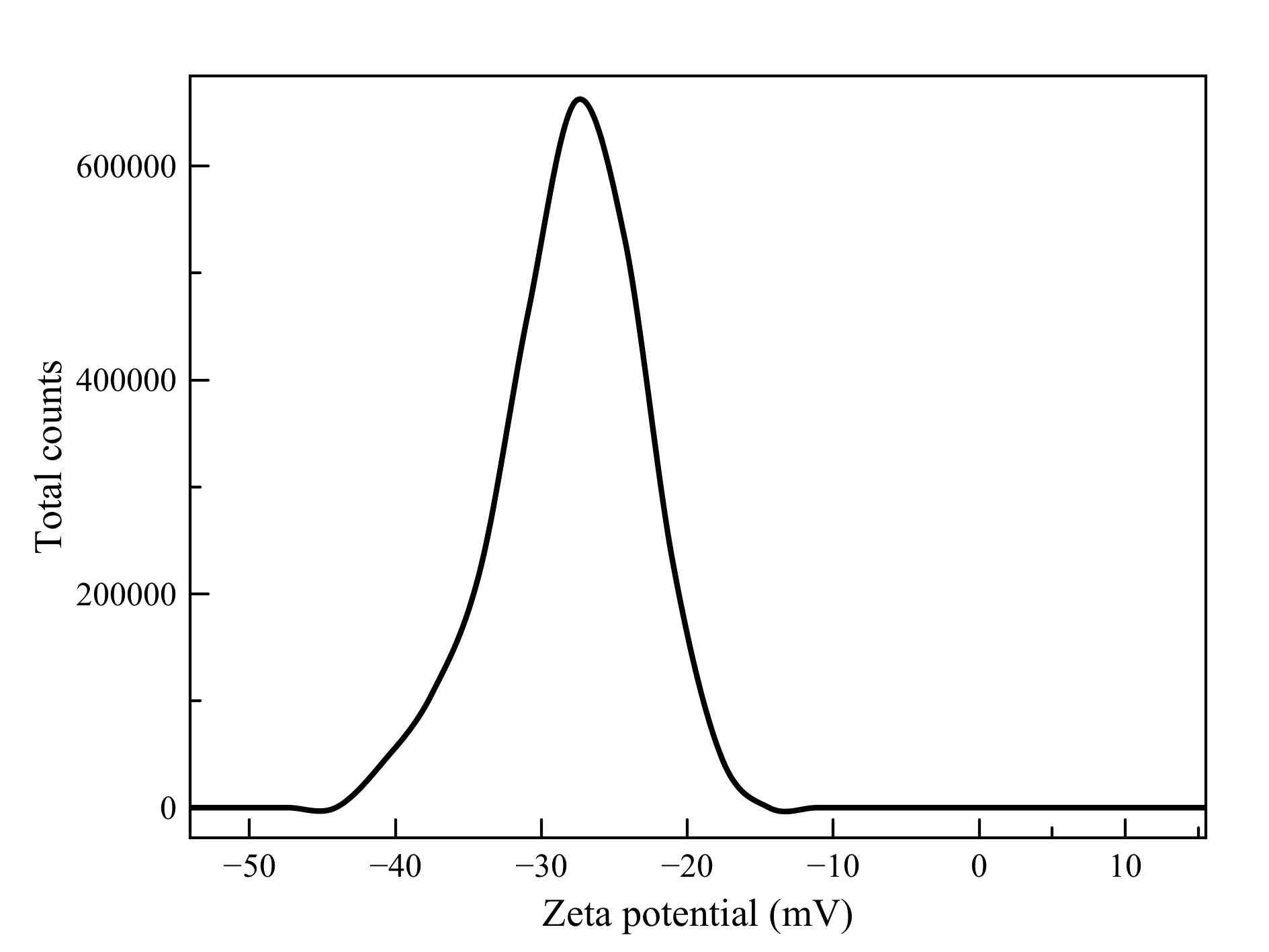
**FIGURE S7** | Zeta potential for PSiNPs in aqueous suspension.


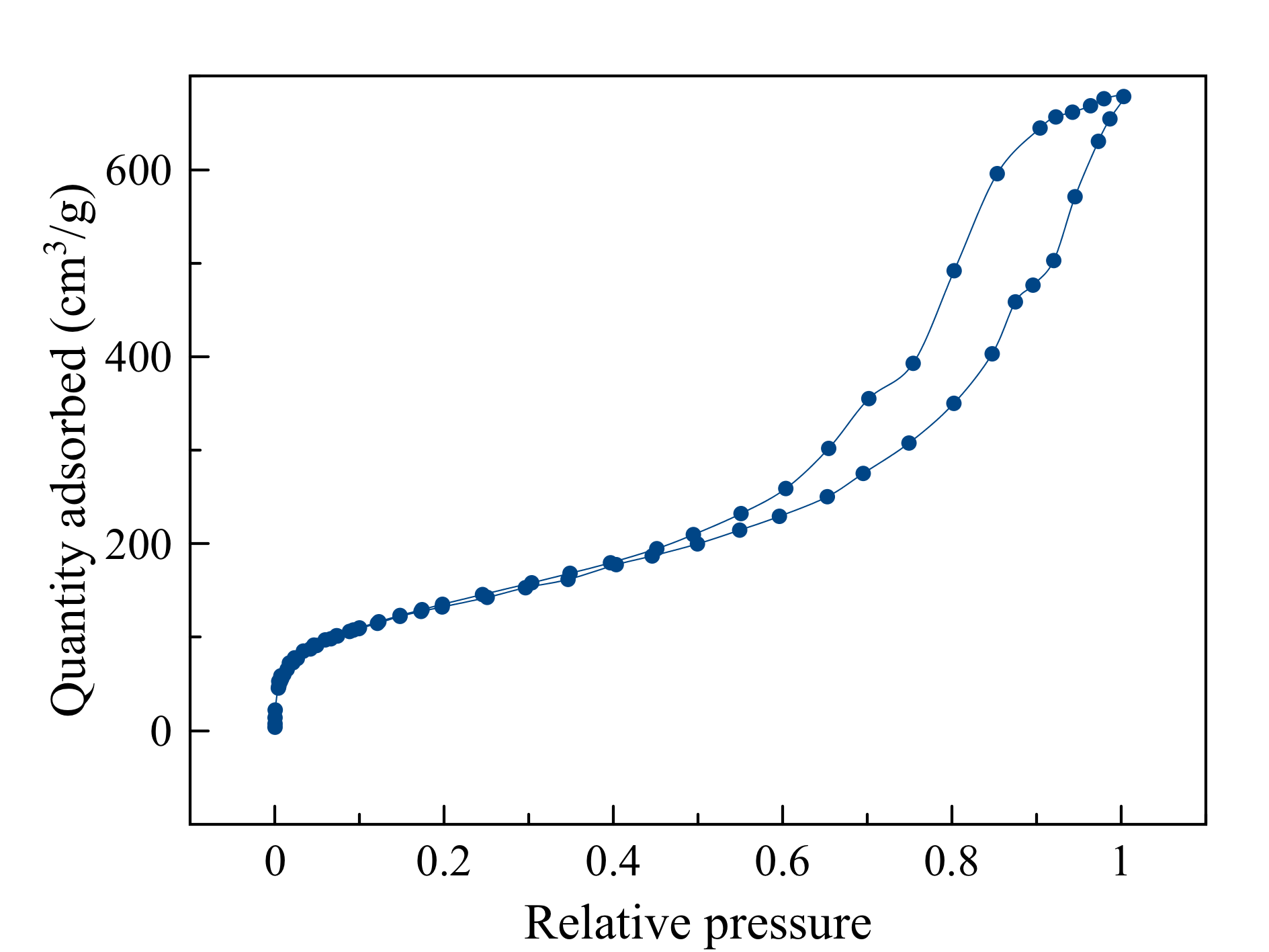


**FIGURE S8** | Low-temperature nitrogen BET/BJH adsorption/desorption isoterms for PSiNPs powders sedimented from aqueous suspensions.


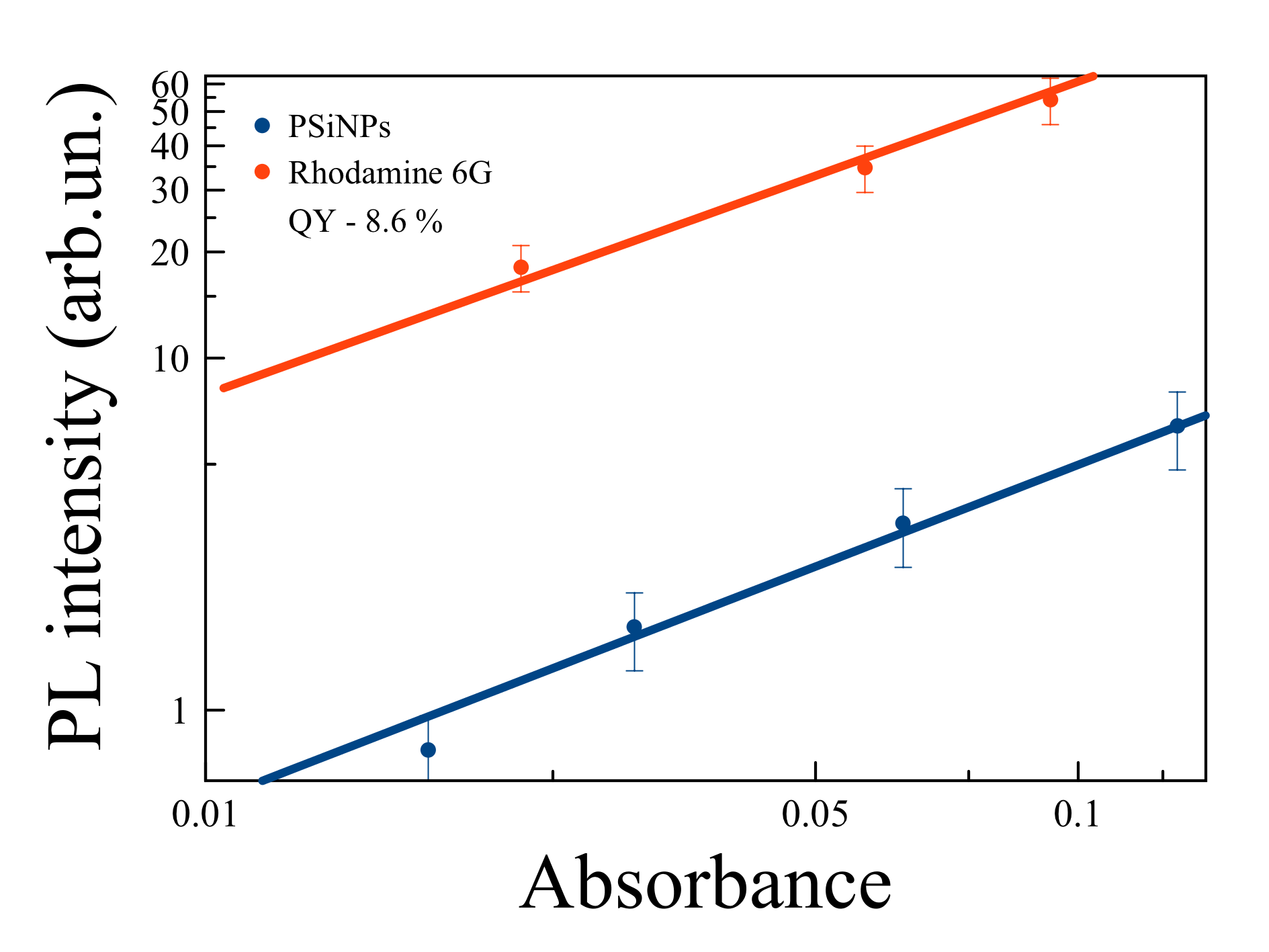
**FIGURE S9** | Example of dependences of PL intensity on absorbance of Rhodamine 6G (red), PSiNPs in aqueous suspension (blue) that were used for calculation of QY of PSiNPs. In this example PSiNPs were oxidized in 0.81 mM of borax for 90 min after termination of CA.


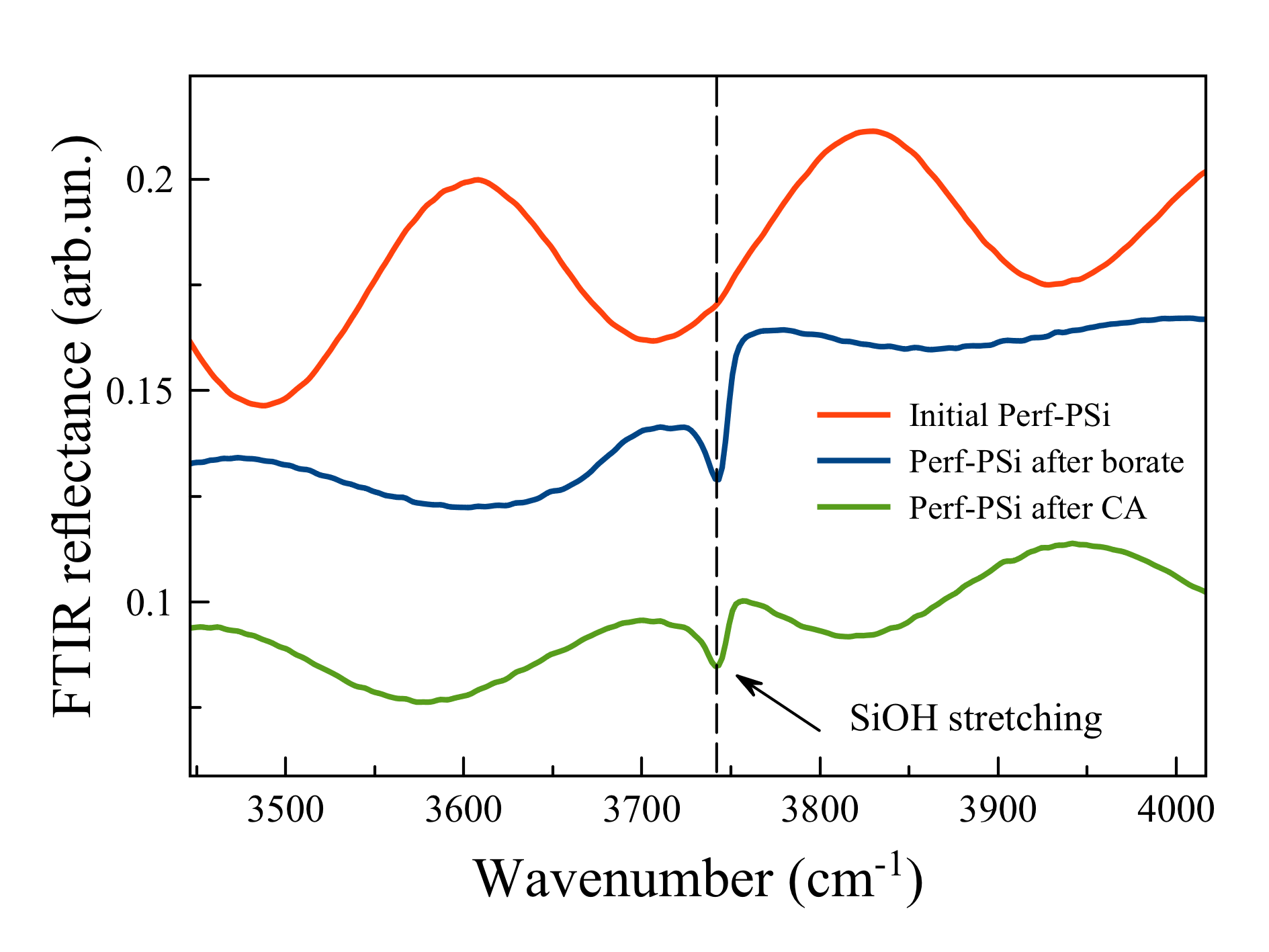


**FIGURE S10** | FTIR reflection spectra for Perf-PSi layers: initial (red), after 20 minutes of oxidation in 13 mM borax solution (rinsed and dried afterwards) (blue), after subsequent 10 minutes of CA treatment (green). SiOH stretching band is pointed using an arrow and a dashed line.

**References:**

Anderson, M. A., Tinsley-Bown, A., Allcock, P., Perkins, E. A., Snow, P., Hollings, M., et al. (2003). Sensitivity of the optical properties of porous silicon layers to the refractive index of liquid in the pores. *Phys. Status Solidi Appl. Res.* 197, 528–533. doi:10.1002/pssa.200306558.

Richter, H., Wang, Z. P., and Ley, L. (1981). The one phonon Raman spectrum in microcrystalline silicon. *Solid State Commun.* 39, 625–629. doi:10.1016/0038-1098(81)90337-9.

Meier, C., Lüttjohann, S., Kravets, V. G., Nienhaus, H., Lorke, A., and Wiggers, H. (2006). Raman properties of silicon nanoparticles. *Phys. E Low-dimensional Syst. Nanostructures* 32, 155–158. doi:10.1016/j.physe.2005.12.030.

Paillard, V., Puech, P., Laguna, M. A., Carles, R., Kohn, B., and Huisken, F. (1999). Improved one-phonon confinement model for an accurate size determination of silicon nanocrystals. *J. Appl. Phys.* 86, 1921–1924. doi:10.1063/1.370988.
